# Intracellular Reactive Oxygen Species Generation Induced by High‐Frequency Ultrasound in Thickness Vibration Mode

**DOI:** 10.1002/advs.202519582

**Published:** 2026-02-21

**Authors:** Kotaro Fujishiro, Satoshi Okada, Filippo Rossi, Takahiro Kuchimaru, Yuta Kurashina

**Affiliations:** ^1^ Graduate School of Engineering Tokyo University of Agriculture and Technology Tokyo Japan; ^2^ Institute of Integrated Research Institute of Science Tokyo Kanagawa Japan; ^3^ Department of Chemistry, Materials and Chemical Engineering “Giulio Natta” Politecnico di Milano Milano Italy; ^4^ Center for Molecular Medicine Jichi Medical University Tochigi Japan; ^5^ Institute of Engineering Tokyo University of Agriculture and Technology Tokyo Japan

**Keywords:** high‐frequency ultrasound, hydroxyl radical, lithium niobate, reactive oxygen species, thickness vibration mode

## Abstract

Reactive Oxygen Species (ROS), which play a critical role in cellular signaling pathways, are one of the chemical products caused by ultrasound irradiation. In general, ROS generation has been ignored in terms of high‐frequency ultrasound (>5 MHz) due to the limitation of cavitation induction. However, does high‐frequency ultrasound not affect the production of ROS in cells? Here, we propose comparing ROS in solution and intracellular ROS generation by ultrasound at high frequency (6.5 MHz) and at a common frequency (1 MHz) in medical ultrasound applications, thereby elucidating the capabilities of high‐frequency ultrasound on cells by ROS production. We discovered that 6.5 MHz ultrasound, as irradiated by a lithium niobate transducer, generates intracellular ROS more than conventional 1.0 MHz ultrasound. The phenomenon of ROS production by high‐frequency ultrasound having high spatial resolution demonstrated the possibility that ultrasound is capable of effectively generating intracellular ROS at the cellular level.

## Graphical abstract

Cell‐sized high‐frequency ultrasound using a lithium niobate transducer, previously ignored in reactive oxygen species (ROS) production, generated intracellular ROS at frequencies higher than conventionally used. The intracellular generation of ROS by cell‐sized high‐frequency ultrasound revealed in this study is expected to be applied to innovative therapies using ROS induced by high‐frequency ultrasound.

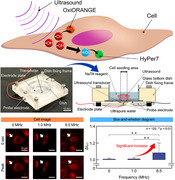

## 1 Introduction

Ultrasound generated by the resonance of objects has a trade‐off relationship between frequency and amplitude, leading to the frequency used varying depending on the desired phenomenon. Reactive oxygen species (ROS), one of the chemical products caused by this ultrasound, play a critical role in cellular signaling pathways, including metabolism [1], aging [2], and cell death signaling by reacting with molecules [3, 4]. Cavitation generated in the liquid phase by ultrasound irradiation provides powerful physicochemical effects, including forming hydroxyl radicals (•OH) and other ROS by pyrolysis of water [5]. Therefore, ultrasound is often used to supply ROS to cells [6]. When ultrasound is applied to a liquid, a cycle of compression and rarefaction occurs, resulting in the phenomenon of acoustic cavitation [7]. When bubbles generated by acoustic cavitation reach the peak of compression, and their size instantly shrinks, localized high‐temperature heat (a few thousand Kelvin) is generated [8]. Localized high‐temperature heat decomposes water molecules, resulting in the formation of ROS [9]. This phenomenon has been used by applying ultrasound at low frequency (∼1 MHz) due to susceptibility to cavitation. Meanwhile, higher‐frequency ultrasound is generally considered to generate little or no ROS. This is because cavitation suitable for ROS generation is less likely to occur at higher frequencies [10]. The size of cavitation bubbles becomes smaller due to the decrease in amplitude as the resonance frequency increases [11], making their growth and collapse less probable. For this reason, ROS generation generally occurs under specific acoustic conditions optimized for sonodynamic or sonochemical applications—not merely by selecting an ultrasound frequency within that range [12, 13, 14]. Consequently, conventional studies on ultrasound‐induced ROS generation have primarily focused on frequencies below 1.0 MHz [15]. For example, 40 kHz (10 W/cm^2^, 60 min) and 1 MHz ultrasound (1.5 W/cm^2^, 50% duty cycles, 10 min) have been used. for tumor treatment. Sensitizers have been used in both experiments. In addition, 40 kHz ultrasound (0.3 W/cm^2^, 10 min) has been used for sterilization or removing harmful substances [15, 16]. Previous studies have shown that the parameters affecting ROS levels are ranked in order of influence: the presence or absence of a sensitizer, frequency, irradiation time, and acoustic pressure [17]. The use of sensitizers largely improves ROS levels, amplifying the cell death rate by several times [18]. Under sensitizer treatment, irradiation with 20 kHz ultrasound at 1 W/cm^2^ for 30 min generated ROS sufficient to induce approximately 50% cell death [17]. This question is whether ultrahigh‐frequency ultrasound is capable of producing enough ROS to affect cells. However, does ultrasound with high frequencies not affect ROS in cells?

**FIGURE 1:**
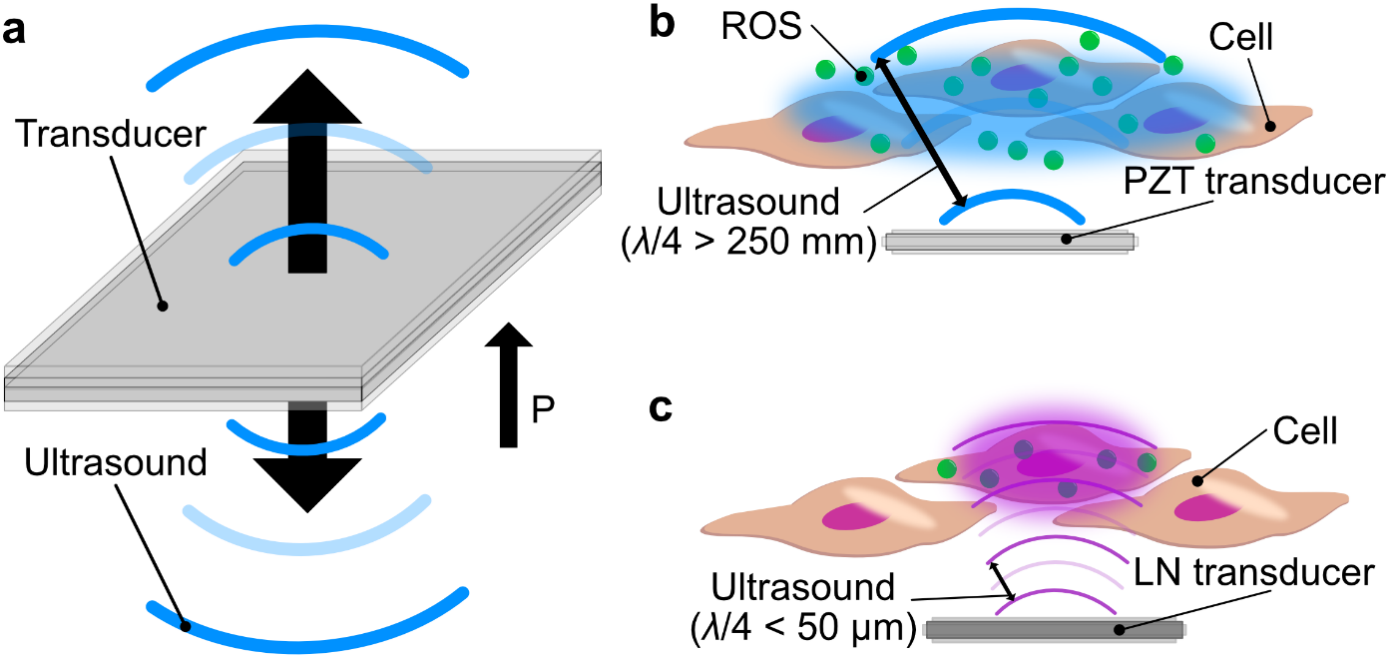
Concept of ROS production by high‐frequency ultrasound irradiation compared with conventional ultrasound irradiation. (a) The mechanism of ultrasound generation by a thickness vibration mode transducer. (b) ROS production by ultrasound irradiation at a conventional frequency (≤1.0 MHz). (c) ROS production by irradiation of high‐frequency ultrasound with a cell‐sized wavelength (*λ*/4 < 50 µm) is proposed in this study.

**FIGURE 2:**
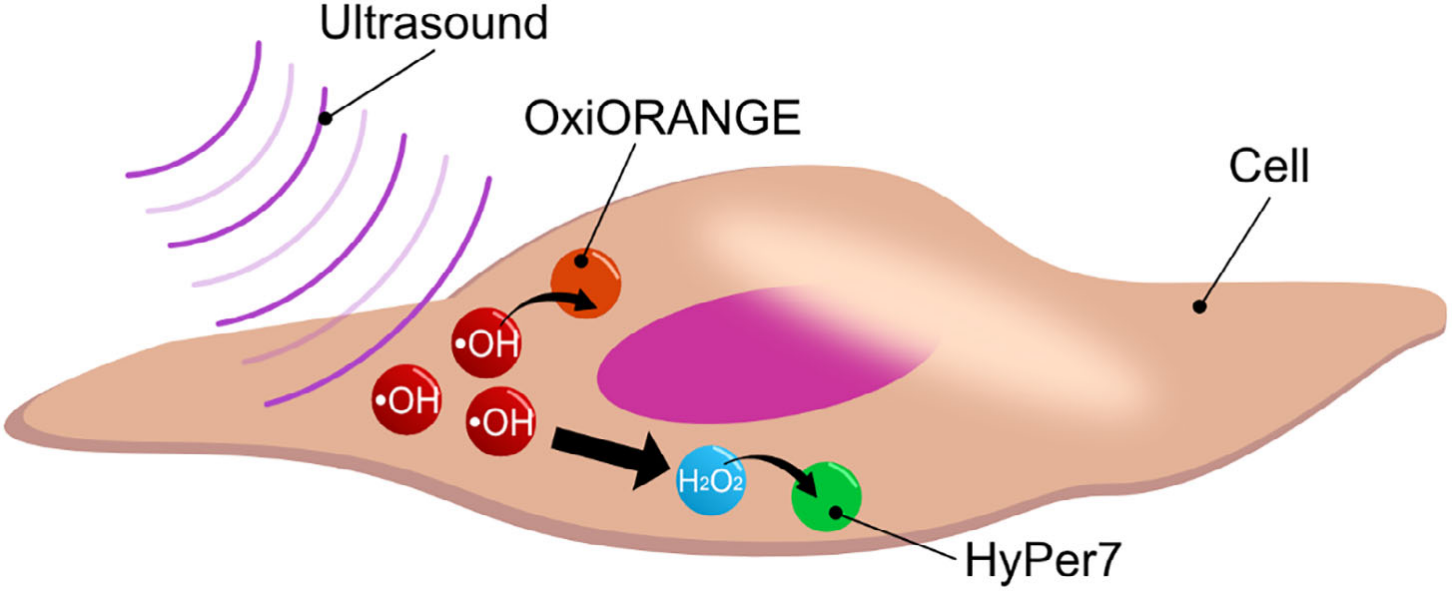
Schematic illustration for measuring intracellular ROS with a fluorescent indicator.

Here, we compare ROS generation by ultrasound at different frequencies inside and outside the cell. Ultrasound irradiation utilized the thickness vibration mode of the transducer (Figure 1a) [19]. Generally, when using a transducer in thickness vibration mode, the waves generated from the transducer become in‐phase waves [20, 21]. Measurement results of ROS generation by ultrasound irradiation showed that high‐frequency ultrasound generates ROS to affect the cell. In particular, the frequency higher than conventional frequencies (≤1.0 MHz, Figure 1b), which is the cell‐sized wavelengths (λ/4 < 50 µm) of a few hundred micrometers, was observed to affect the production of intracellular ROS as opposed to the amount of extracellular ROS (Figure 1c). Such a ROS generation phenomenon was observed because a lithium niobate (LN) transducer with a thickness vibration mode in the high‐frequency region was used for ultrasound irradiation. Ultrasound irradiation using the thickness vibration mode of LN transducers provided high‐power irradiation at cell‐size wavelengths. Cavitation bubbles form at locations where acoustic pressure changes are maximum, so cavitation bubble formation positions depend on the wavelength size [22]. When high‐frequency ultrasound a short wavelength is applied, cavitation bubbles are more easily formed near the bottom surface of the dish, stimulating of cells present at the bottom of the dish [23, 24, 25]. In addition, high‐power ultrasound causes direct mechanical stress to the cells. Mechanical stimulation of cells promotes intracellular ROS generation [26, 27]. ROS generation inside and outside the cell was measured using a fluorescence indicator that changes fluorescence intensity in response to ROS upon exposure to ultrasound of different frequencies. In measuring intracellular ROS generation by ultrasound, •OH generation and its downstream product were targeted (Figure 2). •OH generated by the effect of ultrasound was measured using OxiORANGE (Figure S1a) [28, 29]. In addition, HyPer7 was used to measure hydrogen peroxide (H_2_O_2_) generated downstream of the •OH reaction (Figure S1b). HyPer7 is a fluorescent sensor for detecting H_2_O_2_ [30, 31]. In contrast to ROS generation in solution, high‐frequency ultrasound delivered via LN transducers efficiently generated intracellular ROS compared to conventional ultrasound. Furthermore, two types of fluorescent indicators suggested the visualization of intracellular ROS reactions, partly. That is, the phenomenon of ROS generation even with high‐frequency ultrasound, which was discovered through a multifaceted approach to measuring intracellular ROS and ROS in solution generation, demonstrated the possibility that ultrasound is capable of effectively generating intracellular ROS at the cellular level.

## 2 Results

### 2.1 Fabrication of Ultrasound Device

**FIGURE 3:**
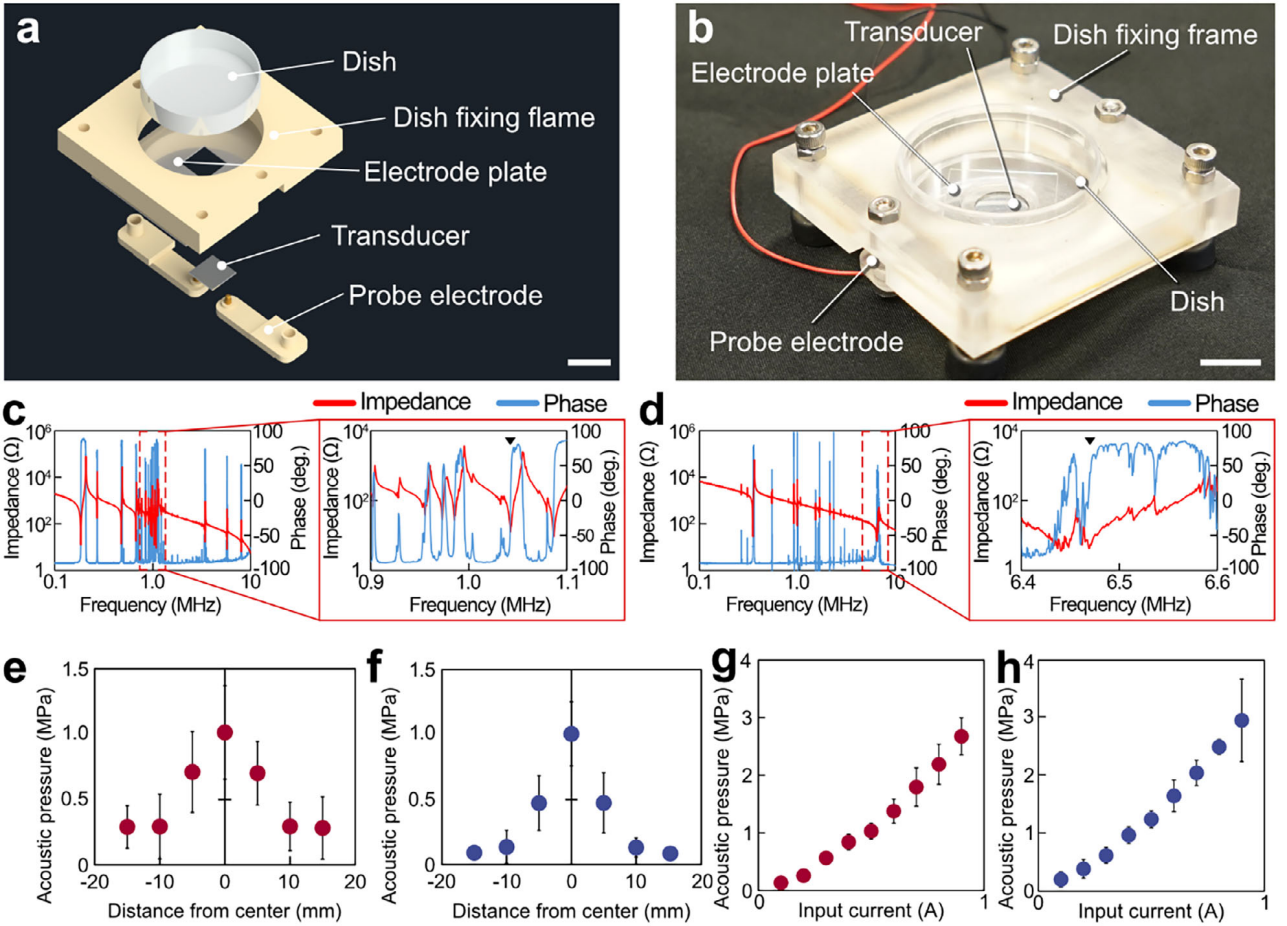
USID with high frequency for supplying ROS to cells. (a) Components and (b) assembly scheme of the ultrasound device. Resonance frequency measurement of ultrasound transducers by an impedance analyzer. (c,d) Impedance and phase were measured in (c) PZT and (d) LN transducers. Expansion of the resonance area in (c) PZT and (d) LN. (e,f) Acoustic pressure distribution in the glass‐bottom dish at (e) 1.0 MHz and (f) 6.5 MHz. (g,h) Relationship between input current and acoustic pressure on the center at resonance frequencies of (g) 1.0 MHz and (h) 6.5 MHz. Scale bars of (a,b) = 10 mm. Error bars of (e–h): mean ± SD, *n* = 10.

For irradiation of high‐frequency ultrasound to cells, an ultrasonic irradiation device (USID) was fabricated to generate ROS in the glass‐bottom dish by high‐frequency ultrasound (Figure 3a,b). This USID was composed of an LN transducer, an electrode plate, a probe electrode, and a dish fixing frame. The LN transducer was fabricated by depositing chromium and aluminum on both sides of the LN wafer in that order and then cutting the wafer into 10 mm squares. Aluminum is deposited so that the wafer is energized. A lead zirconate titanate (PZT) transducer irradiating 1.0 MHz ultrasound was prepared for comparison. PZT electrodes were Ag electrodes on both sides. 1.0 MHz is the frequency used as high frequency in conventional ultrasound for ROS generation [15]. The resonance frequencies of these transducers were measured using an impedance analyzer (Figure 3c,d). Due to the relationship between phase and impedance, the resonance frequencies of the thickness vibration modes with PZT and LN were determined to be 1.0 and 6.5 MHz, respectively. The LN transducer was irradiated with 6.5 MHz ultrasound, which is approximately equal to the quarter wavelength of the cell size. Data such as the size and wavelength of each transducer were summarized (Table 1). The ultrasound transducers were sandwiched between an electrode plate and a probe electrode to activate the transducer by applying an electric current. The glass‐bottom dish was secured with a dish‐fixing frame. The electrode plate was held down by the other electrode probe. The glass‐bottom dish and ultrasound transducer were fixed for constant ultrasound irradiation at the same position and distance. Since the distance between the transducer and the glass‐bottom dish was shorter than the Fresnel zone, a nearly plane wave was irradiated up to the bottom surface of the glass‐bottom dish [32].

**TABLE 1:** Comparison of PZT and LN characteristics.

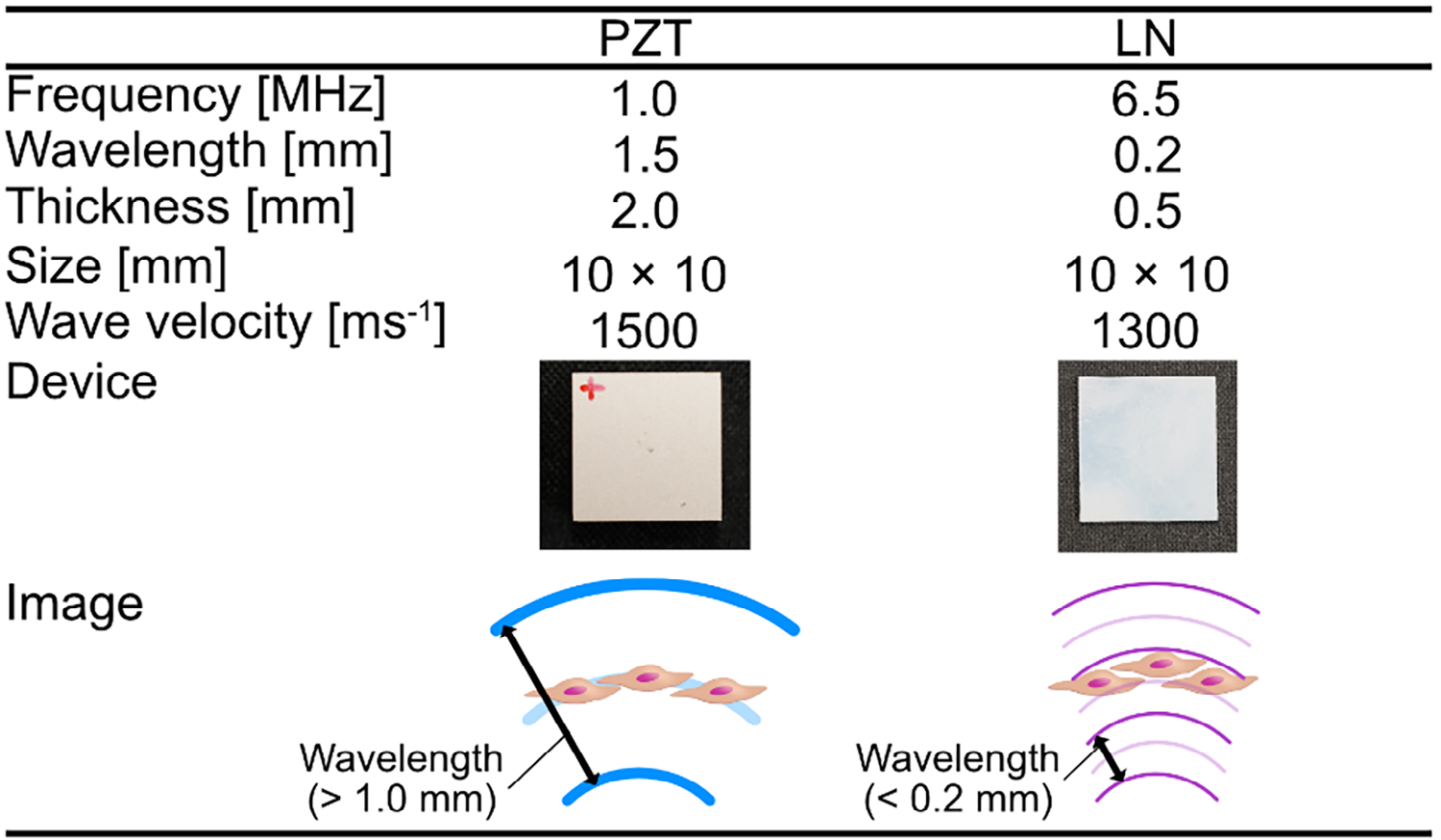

Acoustic pressure distribution was measured by a fiber‐optic hydrophone (Figure 3e,f). For ultrasound irradiation with the LN (Figure 3e) and PZT (Figure 3f) transducers, a mountain‐shaped acoustic pressure distribution was formed with a maximum at the center for both transducers. This is caused by the LN and PZT transducers being structurally constrained by the electrode plate. Comparing the acoustic pressure distribution shapes of LN and PZT transducers, the LN transducer exhibited a sharper acoustic pressure distribution. This was because the attenuation increased as the frequency increased. In addition, the relationship between the input current and the maximum acoustic pressure was measured at a position 1.5 mm from the bottom along the *Z*‐axis at the center of the dish, where the acoustic pressure distribution was at maximum (Figure 3g,h). For both transducers, the acoustic pressure increased linearly with increasing input current.

### 2.2 Evaluation of ROS Generation in Solution by Ultrasound Irradiation

**FIGURE 4:**
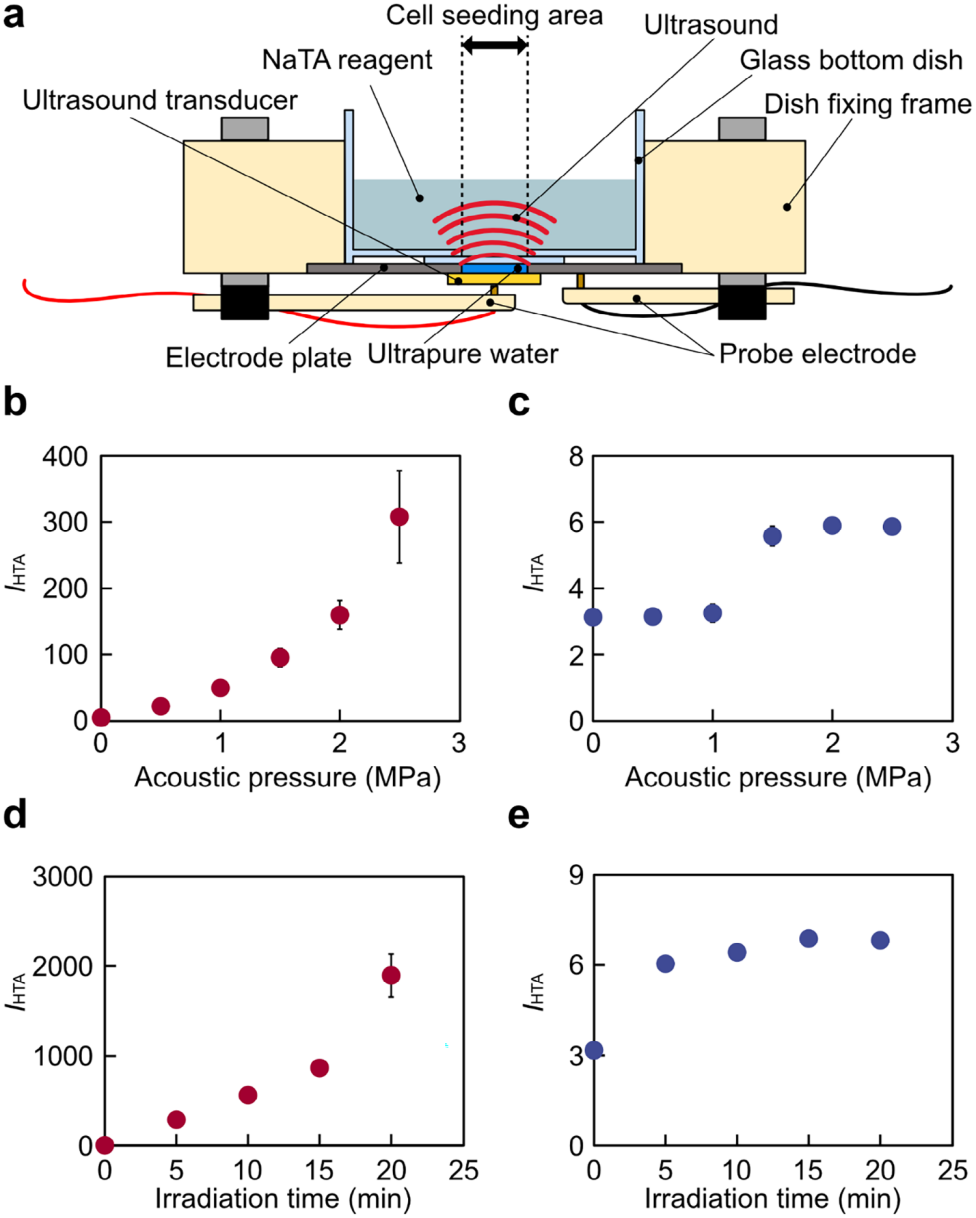
Measurement of changes in ROS generation in solution under various ultrasound irradiation conditions. (a) Experimental setup for ROS generation in solution. (b,c) Relationship between changes in acoustic pressure and fluorescence intensity, *I*_HTA_, at the resonance frequency of (b) 1.0 MHz and (c) 6.5 MHz. (d,e) Relationship between changes in irradiation time and fluorescence intensity, *I*_HTA_, at the resonance frequency of (d) 1.0 MHz and (e) 6.5 MHz. Error bars of (b‐e): mean ± SD. *n* = 3.

For the evaluation of ROS generated in liquids by ultrasound, the amount of ROS generation outside the cells, i.e., in the supernatant in the glass‐bottom dish, was measured (Figure 4a). To measure the change in ROS generation using disodium terephthalate (NaTA) with acoustic pressure, the acoustic pressure varied from 0 to 2.5 MPa at 1.0 and 6.5 MHz, respectively (Figure 4b,c). The fluorescence intensity increased with increasing acoustic pressure at both 1.0 and 6.5 MHz ultrasound. At the resonance frequency of 1.0 MHz, fluorescence intensity increased exponentially with increasing acoustic pressure. At the resonance frequency of 6.5 MHz, fluorescence intensity increased slightly from 1 to 2 MPa of acoustic pressure. This result indicated that even a 6.5 MHz ultrasound generated a small amount of ROS. In addition, to evaluate the effect of irradiation time on ROS generation, the fluorescence intensity of HTA was measured at the resonance frequency of 1.0 and 6.5 MHz, separately, when the irradiation time was changed (Figure 4d,e). The results show that the fluorescence intensity increased with increasing irradiation time at each frequency. At 1.0 MHz, the acoustic pressure increased exponentially with increasing irradiation time, as was the case when the acoustic pressure was varied. On the other hand, at 6.5 MHz, the fluorescence intensity increased significantly from 0 to 5 min and increased gradually at the following times. As in previous studies [33, 34, 35, 36], ROS generation increased more at a lower frequency based on the difference in cavitation generation under both conditions in which the acoustic pressure and the irradiation time were changed (Figure 4b–e). A regression analysis was conducted on the relationship between acoustic pressure and fluorescence intensity at each frequency. The results of the regression analysis showed that for 1 MHz ultrasound, *n* = 18, *p* = 6.3 × 10^−7^, and the effect size was 0.797. Regarding 6.5 MHz ultrasound, *n* = 18, *p* = 5.2 × 10^−7^, and the effect size was 0.802. Similarly, calculating about the irradiation time for 1 MHz ultrasound, *n* = 15, *p* = 4.2 × 10^−7^, and the effect size was 0.869. Regarding 6.5 MHz ultrasound, *n* = 15, *p* = 1.7 × 10^−4^, and the effect size was 0.676. Comparing the results of changing the acoustic pressure and the irradiation time, changing the irradiation time had a dominant effect on the increase in fluorescence intensity for 1 MHz ultrasound. At 6.5 MHz ultrasound, the acoustic pressure had a greater effect than irradiation time.

**FIGURE 5:**
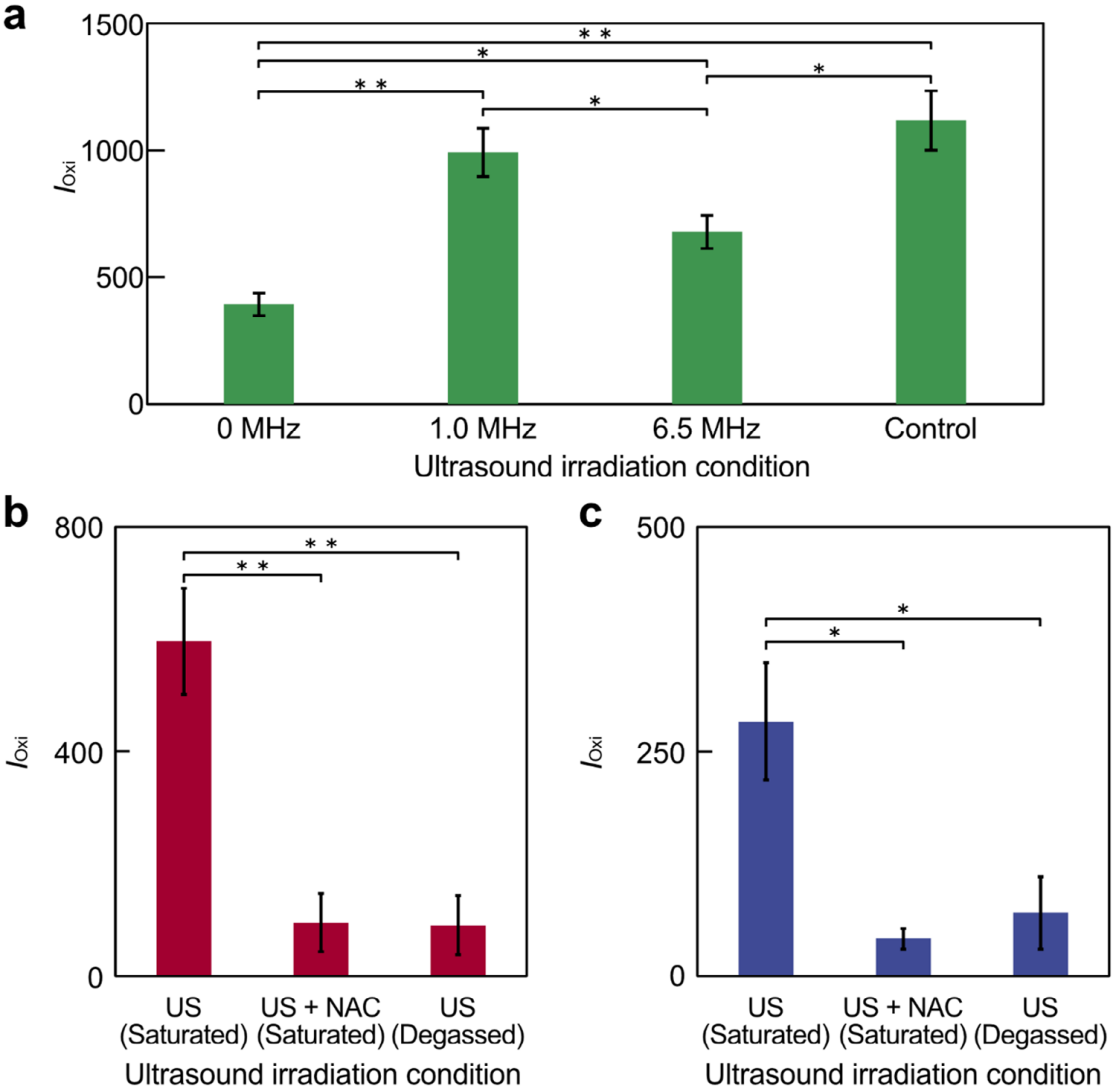
Measurement of ROS generation in solution using OxiORANGE. (a) Fluorescence intensity of OxiORANGE, *I*_Oxi_, in supernatant after ultrasonic irradiation. (b, c) Suppression of ROS generation by ROS scavenger, *N*‐acetyl‐L‐cysteine (NAC), and degassing at (b) 1 MHz and (c) 6.5 MHz. Error bars: mean ± SD. *n* = 3. ^*^ *p* < 0.05, ^**^ *p* < 0.01.

In the observation of intracellular ROS generation with fluorescent probes, OxiORANGE, which was used as a ROS indicator, was evaluated for its reaction to ROS generated in the supernatant as a preliminary stage (Figure 5a). OxiORANGE increases its fluorescence intensity by reacting with •OH, a kind of ROS. Three types of ultrasound irradiation conditions were used: 0, 1.0, and 6.5 MHz. Ultrasound was applied continuously at 2 MPa for 5 min. 500 µm H_2_O_2_ was sprayed as a control condition to check that OxiORANGE reacted to ROS. From three different ultrasound irradiation conditions, fluorescence intensity increased at 1.0 and 6.5 MHz ultrasound, similar to the results with NaTA (Figure 4d,e). At this time, the *MI*, *I*_SPPA_, and *I*_SPTA_ for each frequency were calculated. Regarding 1 MHz ultrasound, *MI* = 1.0, *I*_SPPA_ = 33.3 W/cm^2^, and *I*_SPTA_ = 33.3 W/cm^2^. Regarding 6.5 MHz ultrasound, *MI* = 0.39, *I*_SPPA_ = 33.3 W/cm^2^, and *I*_SPTA_ = 33.3 W/cm^2^. For *I*_SPPA_ and *I*_SPTA_, the calculations were made assuming a free field, but the actual experimental system was not a perfect free field. In addition, water absorption was low. Therefore, the effect of intensity should be much smaller than the calculated value.

Measurements were conducted to confirm that the increase in OxiORANGE fluorescence intensity was caused by the generation of ROS induced by ultrasound irradiation (Figure 5b,c). As experimental conditions, both the OxiORANGE solution containing *N*‐acetyl‐L‐cysteine (NAC) and the degassed OxiORANGE solution were used. In measurements using ultrasound irradiation at any frequency, the fluorescence intensity was significantly reduced under conditions where the ROS scavenger NAC was applied or where degassing was performed. In addition, to evaluate the effect of ultrasound on fluorescent dyes, Rhodamine B, a dye structurally similar to OxiORANGE, was used to confirm that the decomposition of the dye by ultrasound did not contribute to the intensity of fluorescence (Figure S2). In Rhodamine B measurements, the temperature of each sample after irradiation was cooled to the temperature before irradiation. The temperature before irradiation was 24°C. The temperature after irradiation was 36°C in the 1 MHz condition and 32°C in the 6.5 MHz condition. From the measurement results, no change in fluorescence intensity was observed under the irradiation conditions. Therefore, OxiORANGE reacted with •OH generated by ultrasound irradiation.

### 2.3 Evaluation of Intracellularly Generated ROS by Ultrasound Irradiation

The fluorescent probe for detecting •OH was used to evaluate the intracellular ROS generation by ultrasound irradiation with varied acoustic pressure and frequency.

**FIGURE 6:**
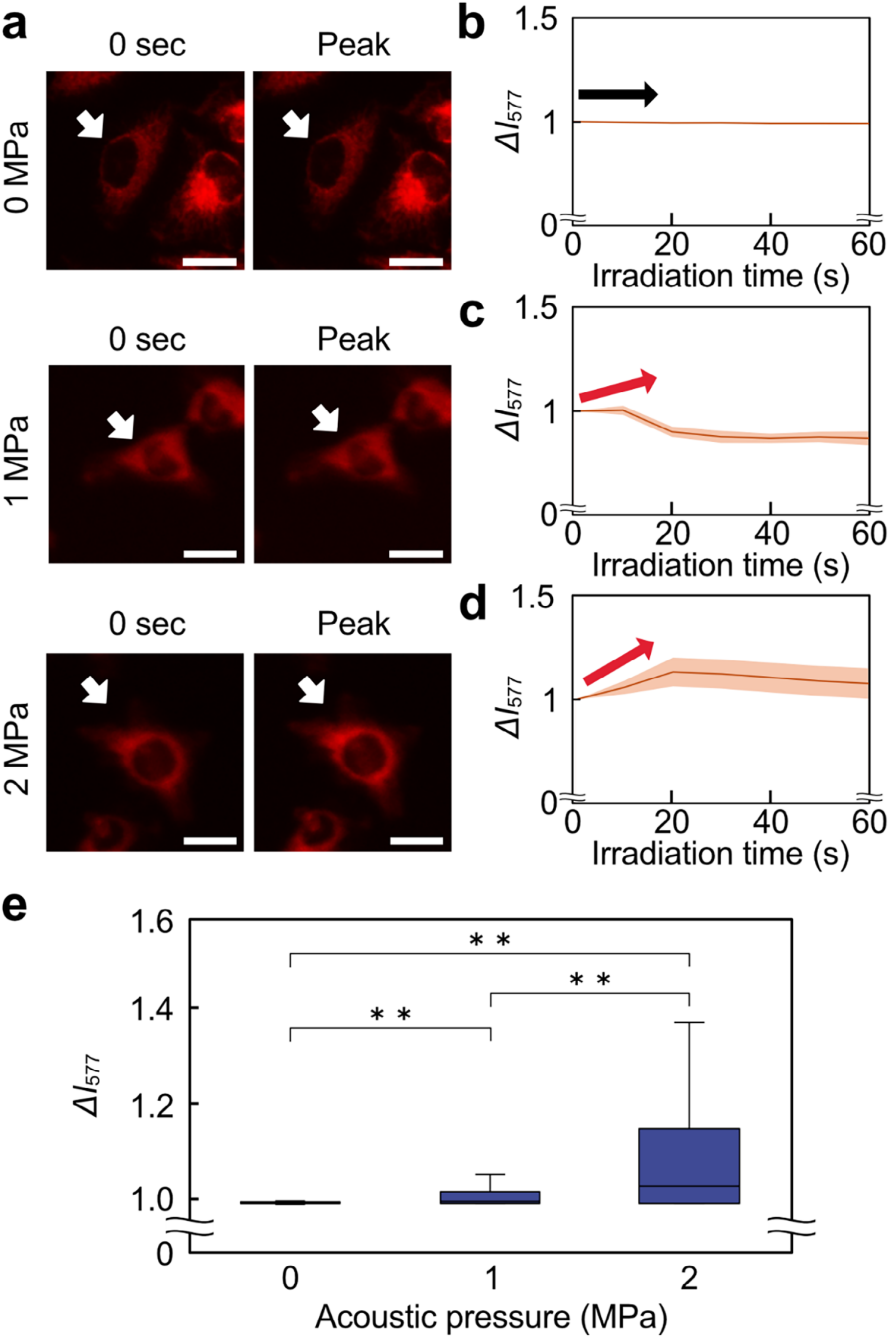
Relationship between acoustic pressure and ROS generation in 6.5 MHz ultrasound irradiation by OxiORANGE. (a) Change in OxiORANGE fluorescence intensity at each acoustic pressure. The white arrows indicate one of the measured cells. (b–d) Time course graph of normalized fluorescence intensity, *ΔI*_577_, at (b) 0, (c) 1, and (d) 2 MPa. The brown lines represented the average line. The orange shades represented the error band for fluorescence intensity. The red and black arrows indicate an increase or decrease in fluorescence intensity. (e) Box‐and‐whisker plots of peak normalized fluorescence intensity for each acoustic pressure condition. Scale bars = 10 µm. Error bars of (e): mean ± SD. *n* = 120. ^**^ *p* < 0.01.

First, the correlation between acoustic pressure and intracellular •OH generation was investigated using OxiORANGE. OxiORANGE fluorescence intensity, *I*_577_, was normalized using the fluorescence intensity at the beginning of the observation. Normalized OxiORANGE was defined as *ΔI*_577._ The fluorescence intensity of OxiORANGE was obtained by time‐lapse observation of fluorescence. From images of cells before and after ultrasound irradiation in each condition (Figure 6a), the change in *ΔI*_577_ was stronger under ultrasound irradiation at an acoustic pressure of 2 MPa than under the other conditions. To quantitatively evaluate the changes in *ΔI*_577_, time‐course histories of *ΔI*_577_ for each condition were compared (Figure 6b–d). Furthermore, the time‐dependent changes in fluorescence intensity for each cell were also visualized (Figure S3). The orange lines represented *ΔI*_577_ changes in each of the 30 cells, and the brown lines represented average values. The time‐course histories of *ΔI*_577_ for each condition show that the change in *ΔI*_577_ increased as the acoustic pressure was increased. The results at 0 MPa showed that the microscope's laser did not contribute to the increase in fluorescence intensity. In addition, the peaks of the time‐course history of *ΔI*_577_ were compared (Figure 6e). *ΔI*_577_ increased under all irradiation conditions and increased with increasing acoustic pressure. Significant increase in fluorescence intensity, especially between 1 MPa and 2 MPa. Therefore, the intracellular •OH generation changes in response to changes in acoustic pressure.

**FIGURE 7:**
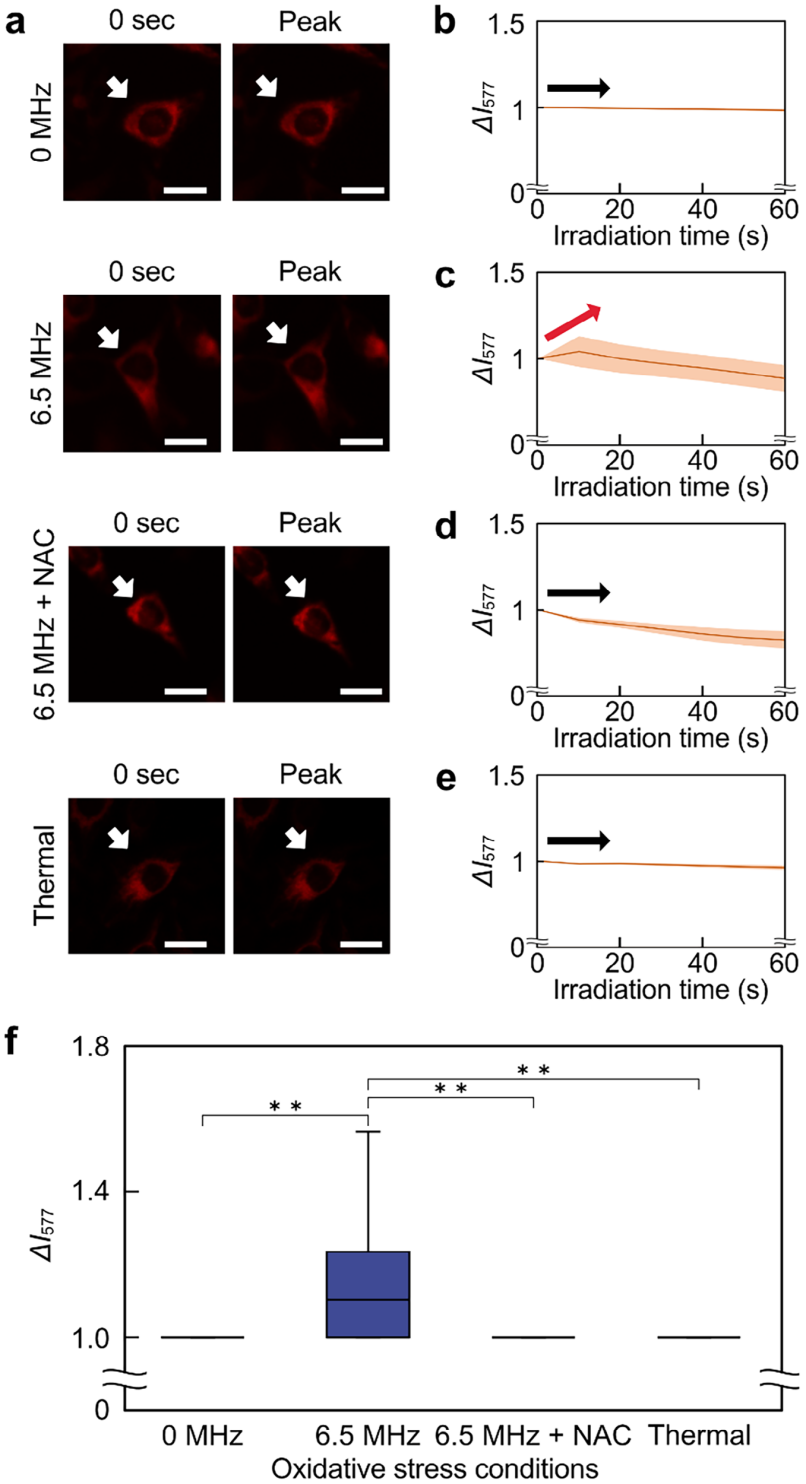
Relationship between oxidative stress conditions and ROS generation by OxiORANGE. (a) Change in OxiORANGE fluorescence intensity at each oxidative stress condition. The white arrows indicate one of the measured cells. (b–d) Time course graph of normalized fluorescence intensity, *ΔI*_577_, at (b) 0 MHz ultrasound, (c) 6.5 MHz ultrasound, (d) 6.5 MHz ultrasound with NAC, and (e) Thermal control. Note that the sample was heated to 32°C, which was the temperature increase caused by the ultrasound. The brown lines represented the average line. The orange shades represented the error band for fluorescence intensity. The red and black arrows indicate an increase or decrease in fluorescence intensity. (f) Box‐and‐whisker plots of peak normalized fluorescence intensity for each oxidative stress condition. Scale bars = 10 µm. Error bars of (e): mean ± SD. *n* = 120. ^**^ *p* < 0.01.

To confirm that fluorescence intensity increased due to ROS generation by ultrasound irradiation, the relationship between *ΔI*_577_ and oxidative stress conditions was measured (Figure 7a). Under 6.5 MHz ultrasound conditions, *ΔI*_577_ increased greatly at the cellular image level. No difference in *ΔI*_577_ under the 0 MHz ultrasound condition without ultrasound irradiation, the condition using NAC with 6.5 MHz ultrasound irradiation, or the thermal control conditions. The thermal control conditions were heated at 32°C, with the solution temperature maintained at the same level as that reached by ultrasound irradiation. To quantitatively evaluate the changes in *ΔI*_577_, time‐course histories of *ΔI*_577_ for each condition were compared (Figure 7b–e). Furthermore, the time‐dependent changes in fluorescence intensity for each cell were also visualized (Figure S4). The orange lines represented *ΔI*_577_ changes in each of the 30 cells, and the brown lines represented average values. From the time‐course histories, the increase in fluorescence intensity was observed under the 6.5 MHz ultrasound irradiation condition. Fluorescence intensity decreased under the conditions with NAC. In addition, the peaks of the time‐course histories of *ΔI*_577_ were compared (Figure 7f). Comparison of the peaks in each condition showed that the fluorescence intensity was significantly increased under the 6.5 MHz ultrasound irradiation condition, while no increase in fluorescence intensity was observed under the other conditions.

**FIGURE 8:**
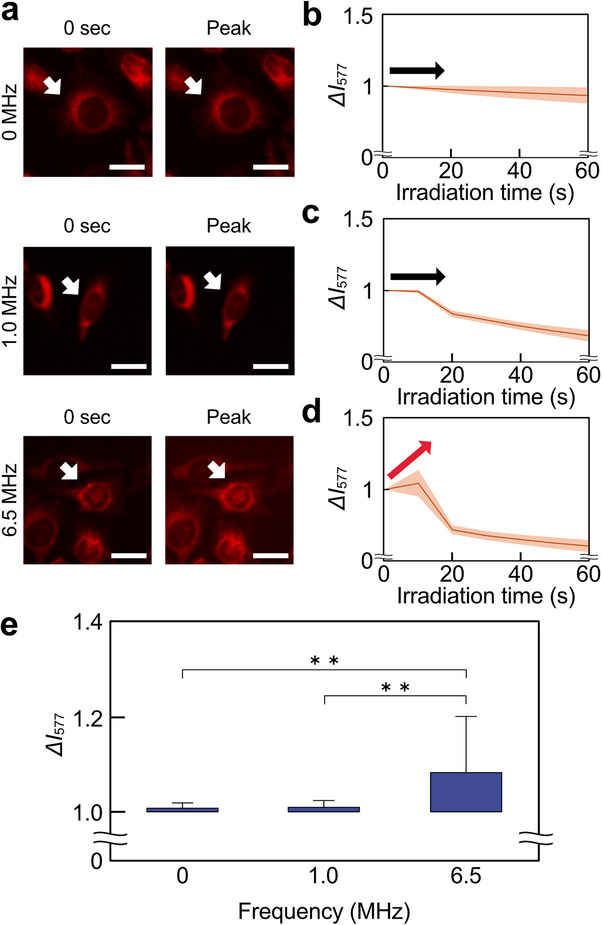
Relationship between frequency and ROS generation in ultrasound irradiation by OxiORANGE. (a) Change in OxiORANGE fluorescence intensity at each frequency. The white arrows indicate one of the measured cells. (b‐d) Time course graph of normalized fluorescence intensity, *ΔI*_577_, at (b) 0 MHz, (c) 1.0 MHz, and (d) 6.5 MHz. The brown lines represented the average line. The orange shades represented the error band for fluorescence intensity. The red and black arrows indicate an increase or decrease in fluorescence intensity. (e) Box‐and‐whisker diagram of peak normalized fluorescence intensity for each frequency condition. Scale bars = 10 µm. Error bars of (e): mean ± SD. *n* = 120. ^**^ *p* < 0.01.

Next, we investigated the effects of ultrasound frequency on •OH generation. Comparison of changes in *ΔI*_577_ with cell images (Figure 8a). Under 6.5 MHz conditions, *ΔI*_577_ changed markedly at the cellular image level. No difference in *ΔI*_577_ between 0 MHz as a control and 1.0 MHz condition. To evaluate the changes in intracellular •OH over time, *ΔI*_577_ was plotted as time‐course histories for each condition (Figure 8b–d). The time‐dependent changes in fluorescence intensity for each cell were also visualized (Figure S5). The orange lines represented *ΔI*_577_ changes in each of the 30 cells, and the brown lines represented average values. These results showed that an increase in *ΔI*_577_ was observed at 6.5 MHz. In addition, the peaks of the time course history of *ΔI*_577_ were compared (Figure 8e). The box‐and‐whisker plots showed that the peak *ΔI*_577_ was significantly increased in the 6.5 MHz condition compared to the other conditions. This indicated that •OH generation at 6.5 MHz was visualized by OxiORANGE.

### 2.4 Evaluation of •OH Chain Reactions Induced by Ultrasound within Cells

H_2_O_2_ is one of the most extensively studied ROS due to its role as an effector molecule that modifies intracellular biomolecules. To clarify whether •OH generated by ultrasound triggers downstream H_2_O_2_ generation within cells, a genetically‐encoded fluorescent sensors. A fluorescent sensor, HyPer7 [33], was expressed in cells under the ultrasound irradiation (Figure 2c).

**FIGURE 9:**
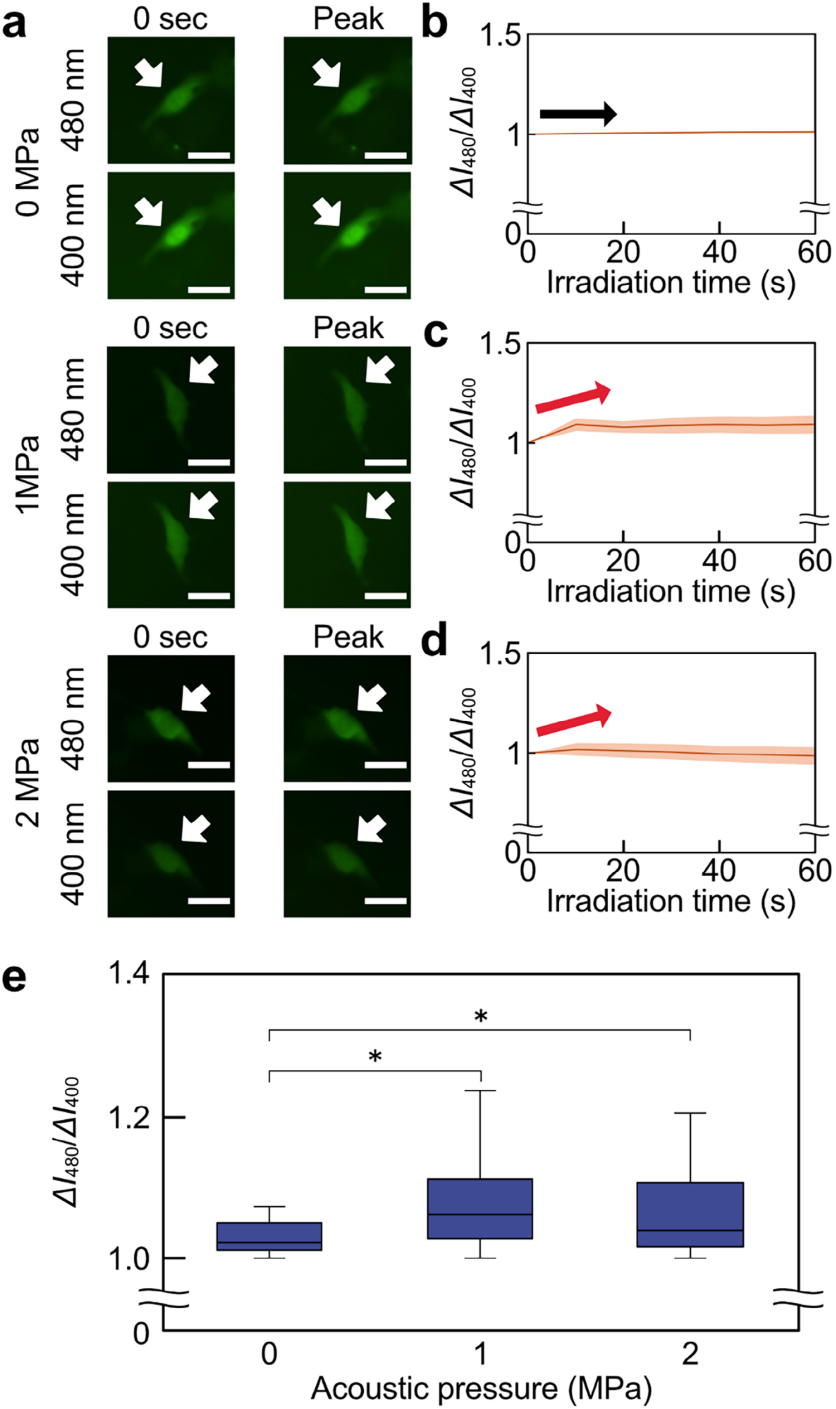
Relationship between acoustic pressure and ROS generation in 6.5 MHz ultrasound irradiation by HyPer7. (a) Change in HyPer7 fluorescence intensity at each acoustic pressure. The white arrows indicate one of the measured cells. (b–d) Time course history of normalized fluorescence intensity ratio, *ΔI*_480_/*ΔI*_400_, at (b) 0, (c) 1, and (d) 2 MPa. The brown lines represented the average line. The orange shades represented the error band for fluorescence intensity. The red and black arrows indicate an increase or decrease in fluorescence intensity. (e) Box‐and‐whisker plots of the peak values of the time course history of *ΔI*_480_/*ΔI*_400_ were shown for each acoustic pressure condition. Scale bars = 10 µm. Error bars of (e): mean ± SD. *n* = 40. ^*^ *p* < 0.05.

Hyper7 successfully visualized intracellular H_2_O_2_ generation induced by 6.5 MHz ultrasound irradiation at different acoustic pressures (Figure 9a). Ultrasonic irradiation at the resonance frequency of 2 MPa resulted in a slight change in a slight *ΔI*_480_/*ΔI*_400_. To evaluate the changes in intracellular H_2_O_2_ over time, *ΔI*_480_/*ΔI*_400_ was plotted as time‐course histories for each condition (Figure 9b–d). The time‐dependent changes in fluorescence intensity for each cell were also visualized (Figure S6). The orange lines represented the fluorescence change for each of the 10 cells, and the brown lines represented the average value. Results under 0 MPa conditions showed that the microscope laser was confirmed not to affect changes in the fluorescence intensity of HyPer7. These results showed that an increase in fluorescence intensity was observed at 1 and 2 MPa. In addition, the peaks in the time‐course history of *ΔI*_480_/*ΔI*_400_ were compared in cells exposed to ultrasonic irradiation of various acoustic pressures at a resonance frequency of 6.5 MHz (Figure 9e). The peak values of *ΔI*_480_/*ΔI*_400_ show that the HyPer7 fluorescence intensity ratio increased under all ultrasound irradiation conditions. On the other hand, no significant difference in the peak of *ΔI*_480_/*ΔI*_400_ was found between the 1 MPa and 2 MPa conditions, indicating that changes in acoustic pressure did not dominate H_2_O_2_ production.

**FIGURE 10:**
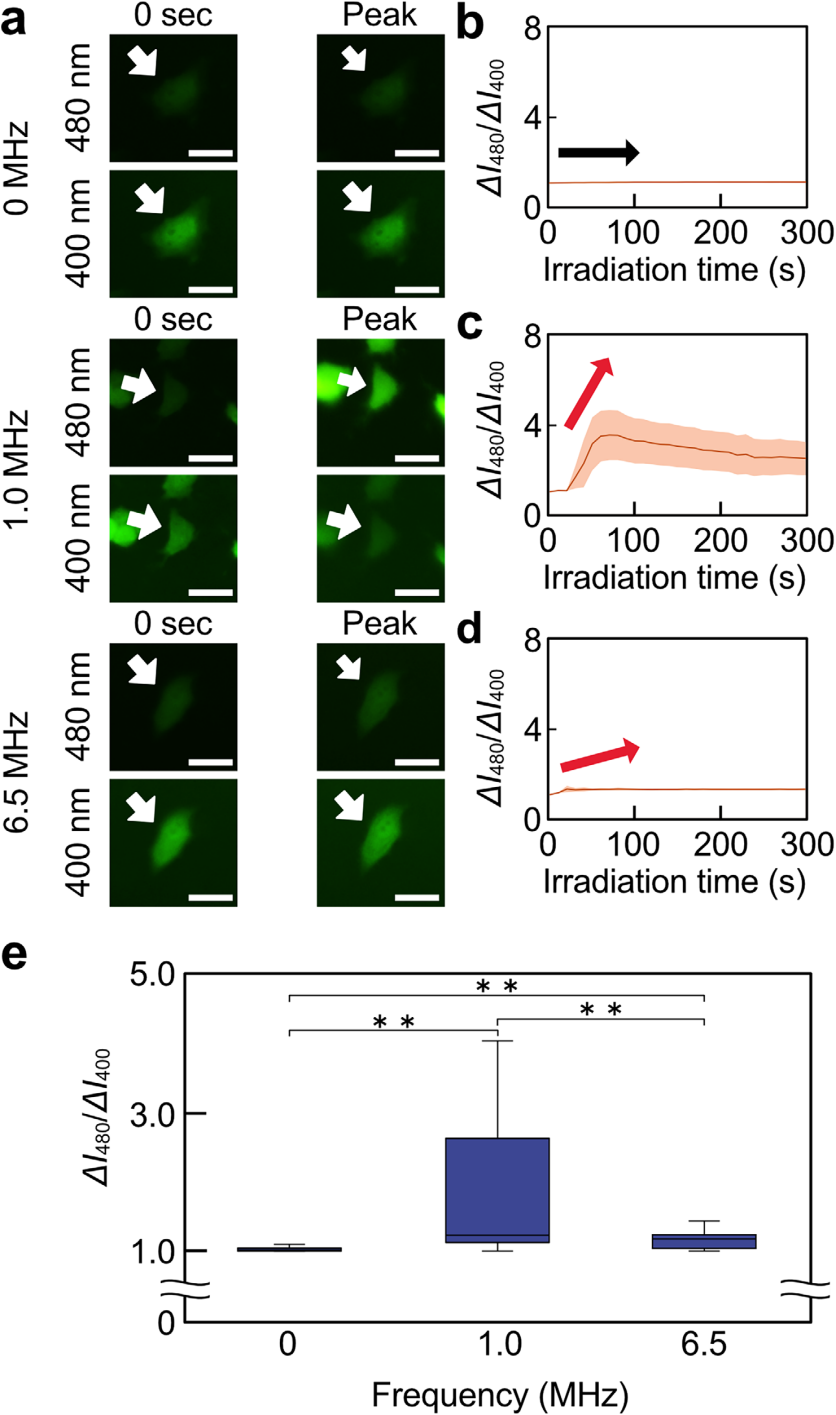
Relationship between frequency and ROS generation in 2 MPa ultrasound irradiation by HyPer7. (a) Change in HyPer7 fluorescence intensity at each acoustic pressure. The white arrows indicate one of the measured cells. (b–d) Time course history of normalized fluorescence intensity ratio, *ΔI*_480_/*ΔI*_400_, at (b) 0, (c) 1, and (d) 6.5 MHz. The brown lines represented the average line. The orange shades represented the error band for fluorescence intensity. The red and black arrows indicate an increase or decrease in fluorescence intensity. (e) Box‐and‐whisker plots of the peak values of the time course history of *ΔI*_480_/*ΔI*_400_ were shown for each acoustic pressure condition. Scale bars = 10 µm. Error bars of (e): mean ± SD. *n* = 40. ^**^ *p* < 0.01.

Additionally, the effect of frequency changes on H_2_O_2_ production was measured, in a manner similar to the •OH generation measurement using OxiORANGE. The fluorescence ratio changes of HyPer7 (*ΔI*_480_/*ΔI*_400_) were monitored as the frequency varied at 0, 1, and 6.5 MHz (Figure 10a). At 6.5 MHz, a modest fluorescence ratio change was detected, while a significant fluorescence ratio change was observed (Figure 10b–d). The time‐dependent changes in fluorescence intensity for each cell were also visualized (Figure S7). The orange lines represented the fluorescence change for each of the 10 cells, and the brown lines represented the average value. These results showed that an increase in fluorescence intensity was observed at 1 and 6.5 MHz. In addition, the peaks in the time‐course history of *ΔI*_480_/*ΔI*_400_ were compared in cells exposed to ultrasound irradiation of various frequencies at an acoustic pressure of 2 MPa (Figure 10e). The peak values of *ΔI*_480_/*ΔI*_400_ showed that the HyPer7 fluorescence intensity ratio increased under all irradiation conditions. Especially, a significant increase in the peak fluorescence intensity ratio was measured at 1 MHz. The increase in fluorescence intensity at 1 MHz indicated that a significant amount of H_2_O_2_ was present within the cells under the irradiation conditions of 1 MHz ultrasound.

### 2.5 Evaluation of Cell Death by Ultrasound Irradiation

**FIGURE 11:**
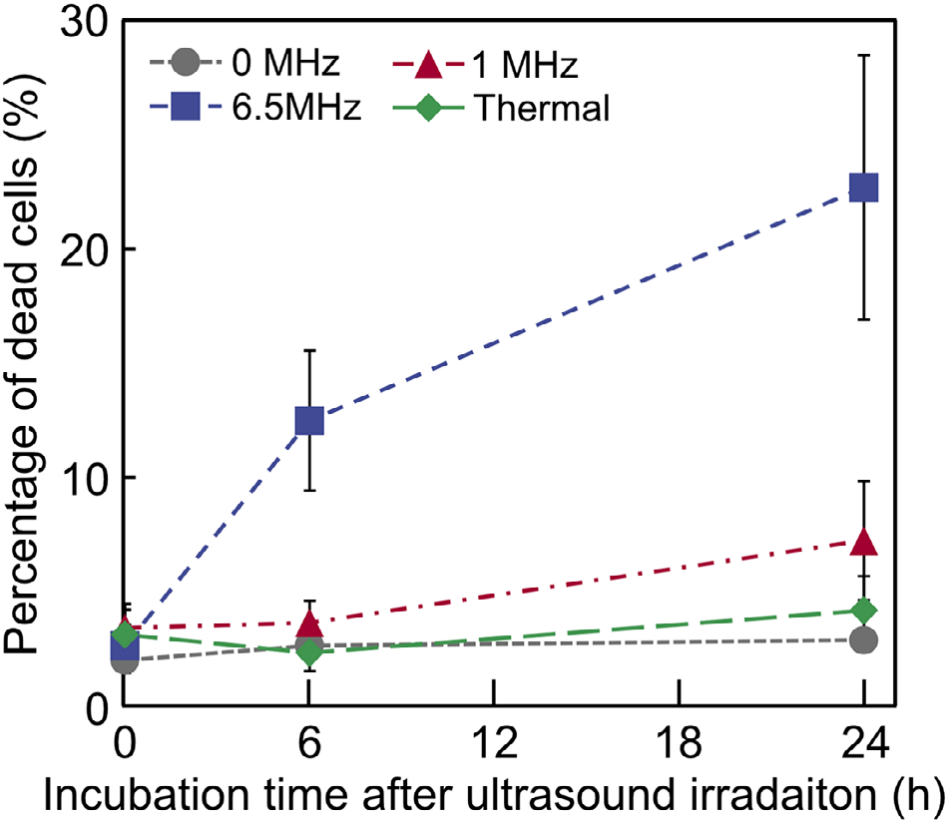
Relationship between incubation time after ultrasound irradiation and percentage of dead cells. Ultrasound at each frequency was irradiated for 5 min at an acoustic pressure of 2 MPa. mean ± SD. *n* = 3.

To evaluate cell viability after ultrasound irradiation, the percentage of cell death after irradiation was measured (Figure 11). The percentage of cell death was calculated from the number of cells stained with Calcein‐AM and PI. Ultrasound was applied at 1 and 6.5 MHz to measure changes in the cell death ratio across frequencies. The 0 MHz ultrasound condition was the cells observed without ultrasound irradiation. In addition, the thermal control condition was provided to evaluate the rate of cell death due to the thermal effects of ultrasound irradiation. The temperature under the thermal control conditions was determined to be 32°C from the temperature increase during irradiation with 6.5 MHz ultrasound (Figure S8). The results showed no increase in the cell death ratio immediately after irradiation across all conditions. At 6 and 24 h after irradiation, the rate of cell death increased in the ultrasound irradiation conditions. In particular, under the 6.5 MHz ultrasound irradiation condition, the change in cell death rate was greatest at 6 h of incubation and increased gradually over the next 24 h.

### 2.6 Evaluation of Intracellular ROS by Ultrasound Irradiation in 3D Tissues

**FIGURE 12:**
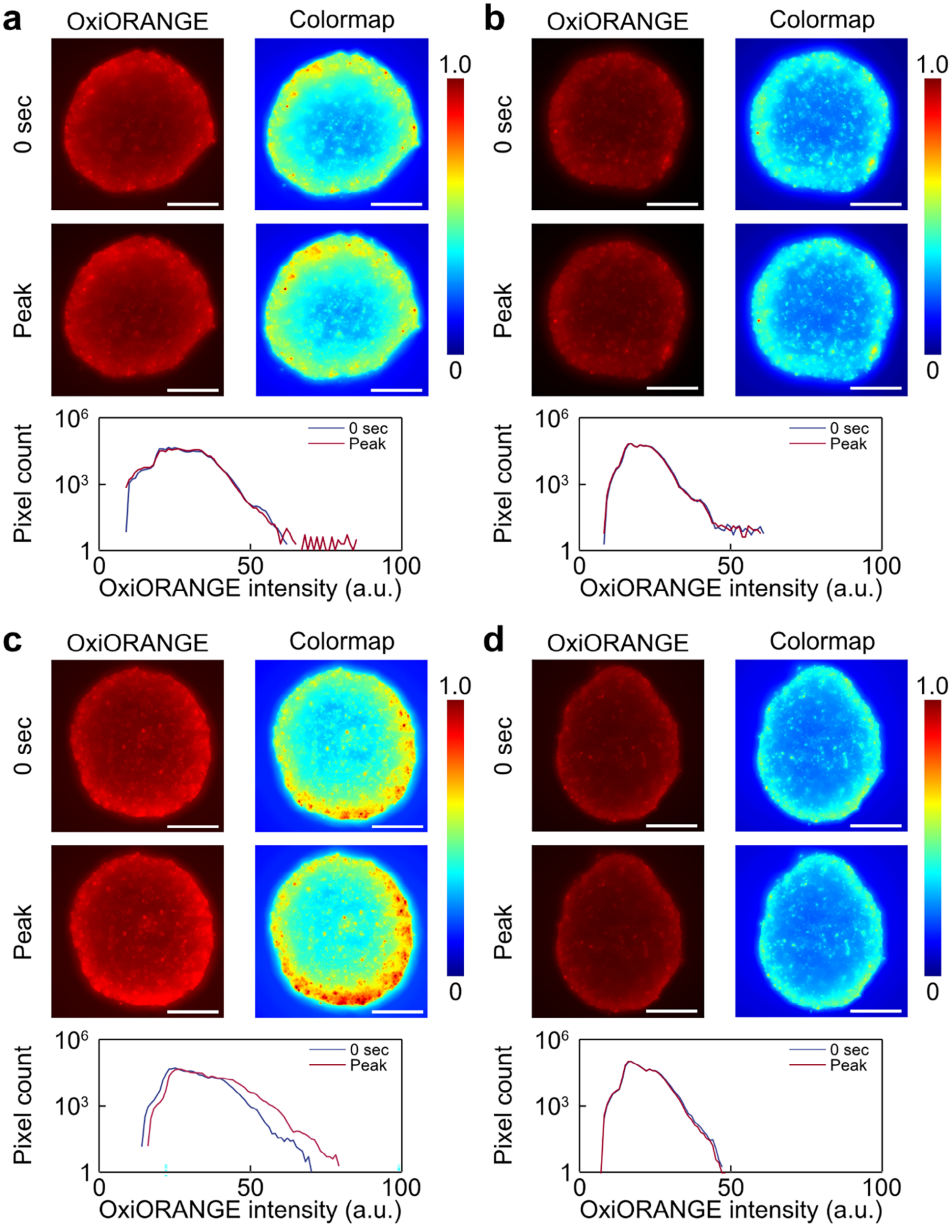
Relationship between oxidative stress and ROS generation in spheroids. (a–d) OxiORANGE images, colormap images, and changes in OxiORANGE fluorescence intensity distribution under oxidative stress conditions at (a) 0, (b) 1, (c,d) 6.5 MHz ultrasound. (d) Ultrasound was irradiated after pretreatment with NAC. Scale bars = 200 µm.

To evaluate intracellular ROS generation induced by high‐frequency ultrasound at the 3D tissue level, ultrasound was irradiated onto spheroids (Figure 12). Spheroids were stained with OxiORANGE to visualize ROS. Spheroid images of the upper half were captured and processed with Z‐stacking. To evaluate the effect of frequency, 0, 1, and 6.5 MHz ultrasound irradiation conditions were prepared. Note that the condition of 6.5 MHz ultrasound irradiation with the addition of NAC was also provided (Figure 12a‐d). The 0 MHz ultrasound condition was the sham ultrasound condition without ultrasound irradiation. For each condition, comparisons were made based on images of OxiORANGE‐stained tissue, their colormap images, and changes in the fluorescence intensity distribution of OxiORANGE during ultrasound irradiation. The results showed that only the condition in which a 6.5 MHz ultrasound was applied exhibited a large change in fluorescence intensity. No change in fluorescence intensity was observed under other conditions, such as irradiation with low‐frequency ultrasound or the ROS scavenging condition. Colormap images showed changes in fluorescence intensity from the underside of the spheroids under 6.5 MHz ultrasound irradiation conditions. In addition, in the fluorescence intensity distribution under the condition of 6.5 MHz ultrasound irradiation, the distribution showed a large rightward shift.

## 3 Discussion

In this study, the generation of ROS in solution and intracellular ROS at 6.5 MHz, which was sufficiently higher than conventionally used frequencies (≤1.0 MHz), was measured. As a result of ultrasonic irradiation at a frequency of 6.5 MHz, even at an acoustic pressure of 2 MPa or more, a small amount of •OH generation was observed outside the cell (Figure 4c,e). On the other hand, ultrasonic irradiation at a frequency of 6.5 MHz to cells generated significant ROS at an acoustic pressure of 2 MPa (Figure 6e). Additionally, •OH generation increased at a frequency of 6.5 MHz compared to 1.0 MHz in experiments with various frequencies of ultrasound irradiation (Figure 8e). These results indicated that 6.5 MHz ultrasound, a higher frequency than conventional transducers, was effective for intracellular •OH generation. Ultrasonic transducers combining high frequency and high output are not available with conventional PZT. Here, we fabricated a USID achieving both high frequency and high output by using the thickness vibration mode of LN (Figure 3a,b). By using the same USID, a thickness vibration mode at a frequency of 1.0 MHz using PZT was driven as a comparison. The resonance frequency was measured for LN and PZT (Figure 3c,d). Here, the results were compared with the calculated resonance frequencies in the thickness vibration mode for LN and PZT. The resonance frequency of the thickness vibration mode was expressed by the following equation.

(1) $$f_{n}=\frac{nv}{2t}$$

At this time, *t* was the thickness of the transducer, and *v* was the acoustic speed in the vibration mode. For LN, where *t* = 0.5 mm and *v* = 7328 m/s, the resonance frequency was 7.33 MHz [37]. For PZT, where *t* = 2 mm and v = 4233 m/s, the resonance frequency was 1.06 MHz [38]. The calculation results were similar to the actual measurement results. ROS generated by ultrasonic irradiation was evaluated in both solution and intracellular environments. This means that the generation of ROS by ultrasound at a frequency of 1.0 MHz, which is conventionally used at high frequencies [39], and the generation of ROS by ultrasound at a frequency of 6.5 MHz proposed in this study were separated into intracellular ROS and ROS in solution generation, demonstrating the diversity of ROS generation.

While ROS generation in high‐frequency ultrasound has conventionally been ignored, 6.5 MHz ultrasound irradiation generated ROS in solution (Figure 4c,e). This ROS generation is indicative of the ROS generation effect of powerful high‐frequency ultrasound irradiation by the LN transducer [21, 40]. The phenomenon of jumping fluorescence intensity observed in both results is thought to be caused by cavitation [10, 41, 42]. This result was confirmed not only in the evaluation of ROS in solution using NaTA but also when the OxiORANGE solution used to visualize intracellular ROS was irradiated (Figure 5a). The difference in the ratio of results between NaTA and OxiORANGE was influenced by differences in the stability and reaction efficiency between HTA and OxiORANGE [43, 44]. In addition, experiments using NAC, one of the ROS scavengers, and a degassed solution showed a decrease in the fluorescent intensity of OxiORANGE (Figure 5). The decrease in fluorescence intensity with the use of NAC indicated that the ROS generation by ultrasound reacted with NAC without reacting with OxiORANGE. The results for the degassed solution indicated that the increase in OxiORANGE fluorescence intensity was due to cavitation. These results indicate that ROS are generated by ultrasound irradiation at a frequency of 6.5 MHz using an LN transducer.

Ultrasound‐induced •OH was measured in cells using the fluorescent indicator OxiORANGE. The trends in •OH generation showed the characteristic effects of frequency and acoustic pressure. OxiORANGE was used to measure the •OH generation in the cells. First, changes in acoustic pressure and •OH generation at 6.5 MHz ultrasound were measured (Figure 6). The fluorescence observed at 0 s of ultrasound irradiation was due to OxiORANGE emitting a certain degree of fluorescence even before irradiation [45, 46]. Furthermore, the decrease or constant in fluorescence intensity after reaching the peak was due to the influence of photobleaching [47]. Additionally, differences in OxiORANGE uptake per cell and variations in intracellular antioxidant levels contributed to increased dispersion under ultrasound irradiation conditions. Therefore, in the experiment, the fluorescence intensity of many cells was measured [48, 49, 50]. A significant increase in •OH generation was observed from 1 MPa to 2 MPa acoustic pressure. The relationship between increased acoustic pressure and •OH suggested that changes in acoustic pressure contribute to the generation of •OH. The effect of temperature changes and using NAC on fluorescence intensity was measured (Figure 7). Changes in fluorescence intensity indicated that thermal control did not affect fluorescence intensity. On the other hand, the use of NAC was suggested to significantly suppress the increase in fluorescence intensity. The two results indicated that the increase in fluorescence intensity in the cells was due to ultrasound‐induced ROS generation. In addition, the effect of different frequencies on •OH generation was measured (Figure 8). Comparison between 1.0 and 6.5 MHz showed that intracellular •OH generation was higher at 6.5 MHz, unlike the measurement of •OH generation in solution. These results indicated that 6.5 MHz ultrasound generated more •OH intracellularly than the conventional frequency of 1.0 MHz. A possible cause of the large amount of •OH in cells at 6.5 MHz is the irradiation with powerful high‐frequency ultrasound in the vicinity of the cells. Powerful irradiation of high‐frequency ultrasound in the vicinity of the cells has reduced the effects of frequency‐dependent attenuation [51]. On the other hand, the lack of •OH generation at 1.0 MHz was attributed to the absence of cavitation around the cells. •OH with short existence lifetimes did not react with OxiORANGE when formed away from the cells, but only when •OH was produced near the cells by irradiation at 6.5 MHz [52, 53]. Additionally, the non‐cavitation effects of 6.5 MHz ultrasound also contribute. The ultrasound induced shear stress via stable cavitation, acoustic streaming, and mechanical stimulation [43, 44]. Shear stress and mechanical stimulation of mechanoreceptors in intracellular organelles contributed to an increase in intracellular Ca^2+^ [26, 54]. Previously, shear stress promoted Ca^2+^ release by stimulating endoplasmic reticulum, mitochondria, and buffer proteins [26, 55]. Mechanical stimulation directly stimulated PANX1 present in the endoplasmic reticulum, causing the release of Ca^2+^ [56]. Furthermore, a minor factor was the mechanical stimulation caused by acoustic streaming. The increase in intracellular Ca^2+^ concentration due to these factors contributed to mitochondrial ROS generation [57]. The factor considered to have caused this phenomenon more frequently than at 1 MHz was the difference in wavelength. High‐frequency ultrasound probably provided a more mechanical stimulus due to a shorter wavelength. Therefore, •OH generation method using high‐frequency ultrasound is effective in realizing highly effective and less damaging treatment methods, such as strong oxidative stress only on the target cells.

In addition, the fluorescent sensor, HyPer7, was used to measure a part of the chain reaction of •OH derived from ultrasound within cells. HyPer7 detected intracellular H_2_O_2_. When irradiated with ultrasonic irradiation at a frequency of 6.5 MHz at different acoustic pressures, *ΔI*_480_/*ΔI*_400_ increased with increasing acoustic pressure (Figure 9). Comparison of time‐course graphs by OxiORANGE (Figure 6b–d) and by HyPer7 (Figure 9b–d) for the change in fluorescence intensity when the acoustic pressure was varied showed a gradual increase with time for HyPer7. These results indicated that the part of the chain reaction of •OH derived from ultrasound was measured by HyPer7. The chain reaction of ROS generated by ultrasound is

(2) $$H_{2}O\;+)))\;\to \;•\mathrm{OH}\;+•H$$

(3) $$•\mathrm{OH}\;+•H\;\to H_{2}O$$

(4) $$•H+O_{2}\to •O_{2}H$$

(5) $$2\;•\mathrm{OH}\;\;\to \;H_{2}O_{2}$$

(6) $$2\;•O_{2}H\;\;\to H_{2}O_{2}+O_{2}$$

(7) $$H_{2}O_{2}+•H\to H_{2}O+•\mathrm{OH}\;$$

where ))) represents ultrasound. H_2_O_2_ is formed downstream of the chain reaction of •OH generated by the effect of ultrasound, as in equations [58]. The time difference between the OxiORANGE time‐course graph and the HyPer7 time‐course graph suggested that the difference was caused by the time delay between the formation of H_2_O_2_ downstream of the reaction. Furthermore, when measuring H_2_O_2_ generation with HyPer7 while fixing the acoustic pressure at 2 MPa and varying the frequency, generation was higher at the 1 MHz condition compared to the 6.5 MHz condition (Figure 10). Despite the low generation of •OH within cells under the condition of 1 MHz ultrasound irradiation, the high measurement of H_2_O_2_ generation indicated that H_2_O_2_ influx from the extracellular environment may be responsible. When 1 MHz ultrasound was irradiated, a large amount of •OH was generated in the solution (Figure 4). These generated •OH were ultimately converted to H_2_O_2_. •OH was highly reactive, whereas H_2_O_2_ was relatively stable with a half‐life of several seconds. Additionally, H_2_O_2_ exhibits high membrane permeability [59, 60]. This was owing to the difference in ROS measured more sensitively by OxiORANGE and HyPer7, which were •OH and H_2_O_2_, respectively. When a 1 MHz ultrasound was irradiated into a NaTA solution, a large amount of ROS was generated (Figure 4b). This indicated that, similar to the chain reaction formula for ROS, •OH generated outside the cell by 1 MHz ultrasound irradiation converted to H_2_O_2_ and then entered the cell.

The state of cells after ultrasound irradiation was evaluated by measuring the percentage of dead cells (Figure 11). The percentage of dead cells immediately after ultrasound irradiation showed little difference relative to the 0 MHz ultrasound condition across all conditions. This result indicated that the cells were not destroyed by the mechanical or thermal effects of the ultrasound. Under 6.5 MHz ultrasound irradiation conditions, the percentage of dead cells increased markedly after 6 h of incubation. Immediately after ultrasound irradiation, few dead cells were observed. The observed cell death after 6 h of incubation was due to ROS generation by the 6.5 MHz ultrasound, which induced apoptosis. This showed a similar trend to the change in the proportion of cells undergoing apoptosis induced by conventional oxidative stress methods [61].

Additionally, ROS generation at the tissue level was evaluated using spheroids (Figure 12). Comparison of fluorescence intensities showed that fluorescence intensity increased under the condition of 6.5 MHz ultrasound irradiation. In addition, under other conditions, little change was observed. From this result, a 6.5 MHz ultrasound was indicated to induce ROS generation even at the tissue level. Colormap imaging confirmed that the fluorescence intensity changed from the outer to the inner regions of the spheroids. This change indicated that at the tissue level, ROS generation‐induced changes were shifting from the bottom to the top in the *Z*‐axis. The use of fluorescence microscopy may have prevented the observation of changes in ROS generation in the deep regions of the spheroids due to reduced light transmission caused by light scattering [62].

Reflecting on the characteristics of high‐frequency ultrasonic irradiation in this study, it suppresses the generation of extracellular ROS and promotes the generation of intracellular ROS, thereby reducing damage to tissues other than target cells. Thus, the generation of intracellular ROS by high‐frequency ultrasound has the potential to produce low‐invasive and effective results in ultrasound‐based cancer treatment. Furthermore, since a quarter of the wavelength is similar to the cell size, improving the spatial resolution of ultrasonic irradiation allows ROS to be generated only in a limited and narrow range of cells. One possibility to increase ultrasound selectivity is to converge 6.5 MHz ultrasound, as in conventional high‐intensity focused ultrasound (HIFU) [63, 64]. To achieve convergent ultrasound like HIFU with LN, a spherical surface formed by polishing or a spherical shape like a soccer ball should be used [65, 66].

## 4 Conclusions

In conclusion, measurement of intracellular ROS and ROS in solution generation using ultrasound of various frequencies showed that cell‐sized high‐frequency ultrasound irradiation using the LN transducer generated more intracellular ROS compared to conventional frequencies. In evaluations of various ROS using the fluorescent indicator and fluorescent sensor, part of the chain reaction of •OH within cells was measured. The intracellular generation of ROS by cell‐sized high‐frequency ultrasound revealed in this study is expected to be applied to innovative therapies using ROS induced by high‐frequency ultrasound.

## 5 Experimental Section/Methods

### 5.1 Preparation of the LN Transducer

A 10 × 10 mm LN transducer was prepared for high‐frequency ultrasound. A 36° Y‐rotated cut LN wafer (Yamaju Ceramics Co., Ltd, JP) was used as the substrate for the LN transducer. The 36° Y‐rotated cut's high durability made it possible to deliver ultrasound at high acoustic pressure [67]. The thickness of the LN wafer was 0.5 mm. To vibrate efficiently without breakage, the thickness of the LN wafer was determined to be 0.5 mm [68, 69]. First, the LN wafer was cut into appropriate sizes and cleaned with isopropyl alcohol (IPA) (166‐04831, Fujifilm Wako Pure Chemical Corporation, OSA, JP). Impurities on the wafer surface were removed by cleaning with IPA. After washing, IPA was removed with water and dried. After drying, the wafer chip was placed in a vacuum evaporator (VE‐2013, Vacuum Device Inc., JP), and both sides were evaporated in sequence, first chrome (CR‐090161, The Nilaco Corporation, JP) and then aluminum (AL‐011565, The Nilaco Corporation, JP). Aluminum chips of the same size were all deposited in a tungsten basket (B‐010, The Nilaco Corporation, JP) set to achieve the same thickness of aluminum on both sides. LN wafer is deposited on both sides and cut on all four sides to make a transducer. By cutting the four sides, the aluminum deposited on the sides was removed and insulated to function as a transducer.

### 5.2 Construction of Ultrasound Irradiation Device

The ultrasound device consisted of a dish with a frame, an electrode plate, a transducer, and probe electrodes. The ultrasound irradiation device comprised a piezoelectric element sandwiched between a probe electrode and an Iron plate (SCB‐05‐3040, Trusco Nakayama Corporation, JP). These parts were fixed with bolts and nuts. Ultrapure water was used to couple the glass‐bottom dish (D11130H, Matsunami Glass Ind., Ltd, JP) and the transducer. A coupling agent was not used. Dish fixing frame and probe electrodes were fabricated by a 3D printer (Form3, Formlabs, MA, US). In 3D printing, resin (Formlabs Clear Resin Cartridge v4, RS‐F2‐GPCL‐04) was used for shaping. The resin consists of urethane dimethacrylate (55–75 (Wt. %), CAS:72869‐86‐4), Methacrylic acid, monoester with propane‐1, 2‐diol (15‐25 (Wt. %), CAS:27813‐02‐1), and diphenyl (2, 4, 6‐trimethylbenzoyl) phosphine oxide (< 0.9 (Wt. %), CAS:75980‐60‐8). The heat deflection temperature of the resin after post‐curing was 58°C at 1.8 MPa and 73°C at 0.45 MPa. The probe electrode was made to function as an electrode by inserting a spring probe into the modeled structure. The electrode plate was fabricated by machining a 0.5 mm‐thick metal plate with a wire electrical discharge machine (AQ327L, Sodic, YOK, JP).

The resonance frequency of the fabricated ultrasound irradiation device was measured using an impedance analyzer (FRA51615, NF Corporation, YOK, JP). Two measurements were performed: 10 × 10 PZT (Z1T36*50W‐S (C‐213), Fuji Ceramics Corporation, JP) and 10 × 10 LN. The impedance analyzer was configured to measure impedance (Ω) and phase (deg) with a sweep frequency range of 100 kHz to 10 MHz, and a sweep resolution of 5000 logarithmic steps. Resonance areas were also measured in linear steps, respectively, by magnifying. Operating the USID at the determined frequency resulted in the highest current values.

During ultrasound irradiation, the frequency and input voltage were controlled by a function generator (WF1947, NF Corporation, YOK, JP), and the input voltage was amplified by a MHz amplifier (LZY‐22+, Mini‐Circuits, NY, US). Furthermore, measuring the voltage and current with an oscilloscope (TBS2074B, Tektronix, OR, US) confirmed that the ultrasound irradiation device was working properly. The voltage was measured using a passive voltage probe (TPP1000, Tektronix, OR, US), and the current was measured using an AC probe (CT2, Tektronix, OR, US). These probes could be measured by connecting them to the oscilloscope.

The fiber‐optic hydrophone (HFO‐690, Onda Corporation, CA, US) was used to measure acoustic pressure. The spatial resolution of the fiber‐optic hydrophone was 100 µm, enabling high‐precision measurement of acoustic pressure. Acoustic pressure measurements were taken with both PZT and LN. Acoustic pressure distribution measurements were taken at 5 mm intervals within a ±15 mm range radially on the *XY*‐plane. For the *Z*‐axis, acoustic pressure was measured with the sample positioned 1.5 mm above the bottom. The relationship between current and acoustic pressure was measured from 0.1 to 0.9 A at the center of the glass‐bottom dish. All measurements were made at peak‐to‐peak values. When measuring acoustic pressure, the optical fiber was mounted on a triaxial motorized stage and measured at a constant height. The glass‐bottom dish was placed in the same position for all experiments.

### 5.3 Measurements of ROS in Solution

NaTA (T1097, Tokyo Chemical Industry Co., Ltd., JP) was used to measure the amount of ROS generated by ultrasound. Hydroxy terephthalic acid (HTA) was formed when NaTA reacted with ROS. HTA was a fluorescent substance with an excitation wavelength of 310 nm and a fluorescence wavelength of 425 nm. The generation of ROS was evaluated by measuring the fluorescence intensity of this substance. The NaTA solution used was prepared by dissolving NaTA powder in water at a final concentration of 1 mm. The NaTA solution was poured 4 mL into a glass‐bottom dish set in the ultrasound device. The amount of ROS generated was evaluated by irradiating the NaTA solution with ultrasound and measuring the fluorescence intensity of the supernatant with a spectrofluorometer (FP‐8550, JASCO Corporation, TYO, JP). The supernatant was the liquid after ultrasound irradiation, not degassed water. Fluorescence intensity for each sample was defined as the fluorescence intensity at 425 nm. The ultrasound frequencies were compared at 1 and 6.5 MHz. In ultrasound irradiation, the acoustic pressure and irradiation time were used as parameters. In experiments with varying acoustic pressure, the acoustic pressure varied from 0 to 2.5 MPa in 0.5 MPa intervals. In this measurement, the ultrasound irradiation time was fixed at 5 min. Similarly, experiments with varying irradiation time was varied from 0 to 20 min at 5‐min intervals. The acoustic pressure was fixed at 2 MPa. In all measurements, ultrasound was continuously irradiated.

OxiORANGE (GC3004‐01, Goryo Chemical, SPK, JP), a ROS‐responsive fluorescent probe used intracellularly, also measured ROS in solution levels. First, OxiORANGE was diluted in PBS (−) to prepare a 0.05 µm solution (Table S1). To align the experimental conditions for measuring ROS generation in solution with and without cells, OxiORANGE was dissolved in PBS (−). 4 mL of the prepared solution was poured into a glass‐bottom dish and irradiated. After irradiation, 1.5 mL of the solution was collected to measure with a spectrofluorometer. Since OxiORANGE has an excitation light of 532 nm, the collected samples were irradiated with 532 nm excitation light. Fluorescence intensity for each sample was defined as the fluorescence intensity at 577 nm. Samples were prepared with 500 µm H_2_O_2_ (081‐04215, Fujifilm Wako Pure Chemical Corporation, OSA, JP), 1.0 MHz ultrasound, 6.5 MHz ultrasound, and static conditions. H_2_O_2_ reacted with OxiORANGE through various mechanisms, such as •OH generation via self‐decomposition and direct reaction, so H_2_O_2_ was used to confirm that OxiORANGE functions [70].

To confirm that the increase in OxiORANGE fluorescence intensity was caused by ROS generated by ultrasound irradiation, the ROS scavenger and the degassed solution were prepared. NAC (013‐05133, Fujifilm Wako Pure Chemical Corporation, OSA, JP) was used as a ROS scavenger. First, NAC was dissolved in 1 mm PBS (−) (09‐8912‐100, TOHO KK., JP). OxiORANGE was diluted to a concentration of 0.05 µm in PBS (−) containing dissolved NAC. Degassed OxiORANGE solution was prepared by diluting OxiORANGE to a concentration of 0.05 µm in PBS (−) and then degassing the solution for 5 min. 4 mL of the prepared solution was poured into a glass‐bottom dish and irradiated. The ultrasound irradiation conditions were 6.5 MHz, 2 MPa, for 5 min. After irradiation, 1.5 mL of the solution was collected to measure with a spectrofluorometer.

In addition, to evaluate the sonobleaching of fluorescent dyes, Rhodamine B (R0040, Tokyo Chemical Industry Co., Ltd., JP) was subjected to ultrasound irradiation experiments. First, Rhodamine B was diluted in PBS (−) to an equivalent concentration of 0.05 µm to that of OxiORANGE diluent. After dilution, 4 mL of the Rhodamine B diluent was poured into a glass‐bottom dish. A glass‐bottom dish containing a diluted solution was set in the ultrasound device and irradiated. Under the ultrasound irradiation conditions, ultrasound at 1 and 6.5 MHz was used. The acoustic pressure was set to 2 MPa under all irradiation conditions. After irradiation, the solution was cooled to the original liquid temperature. This was due to the temperature dependence of Rhodamine B [71]. In addition, the condition for spraying 100 µm H_2_O_2_ was provided. Samples subjected to oxidative stress were measured with a spectrofluorometer. The excitation and fluorescence wavelengths of Rhodamine B were 561 nm and 580 nm, respectively, and the fluorescence intensity at 580 nm was measured.

### 5.4 Preparation of HeLa Cells

HeLa cells (RCB0007, RIKEN BRC CELL BANK, Tsukuba, JP) were cultured in E‐MEM (051‐07615, Fujifilm Wako Pure Chemical Corporation, OSA, JP) in T25 flasks (130189, Thermo Fisher Scientific, MA, US) (Table S2). The medium contained 10% fetal bovine serum (S1600‐500, Biowest Inc., FR). Cells were incubated at 37°C in a humidified atmosphere containing 5% CO_2_. Cell passaging was performed approximately every 3 days.

### 5.5 Measurements of Intracellular ROS by Fluorescent Probe

OxiORANGE, a ROS‐detecting probe, was used to visualize intracellular ROS generation by ultrasound. This probe reacts with various ROS, exhibiting more strongly with •OH and hypochlorous acid (HCLO) to emit orange fluorescence. First, PBS (+) (02492‐94, Nacalai Tesque Inc., JP) was prepared as a buffer to dilute the fluorescent probe. PBS (+) having divalent cations was used to prevent cells from detaching during the uptake of OxiORANGE into cells [72]. PBS (+) was prepared by diluting PBS (+) concentrate 1/100 with PBS (−). Prepare a 0.5 µm solution of the fluorescent probe by diluting it with PBS (+). The medium and diluent were replaced in a glass‐bottom dish in which 2.0 × 10^4^ cells were seeded to allow OxiORANGE to enter the cells. After filling with diluent, the glass‐bottom dish in which the cells were seeded was incubated (37°C, 5% CO_2_) for 20 min. After incubation, cells were washed three times with PBS (−). After washing, 4 mL of PBS (−) was added, and the microscope (ECLIPSE Ni‐E, Nikon Solutions Corporation, TYO, JP) was used to observe cells under different conditions. All fluorescence changes of OxiORANGE in the cells were observed in a time‐lapse. In the experiments using OxiORANGE, four independent experiments were conducted, each measuring the fluorescence intensity of 30 cells.

### 5.6 Measurement of Intracellular ROS by Fluorescent Sensor

Intracellular H_2_O_2_ generation was measured by HyPer7. HyPer7 is characterized by a change in fluorescence intensity at two wavelengths when oxidized by H_2_O_2_. The fluorescence wavelength with an excitation wavelength of 400 nm is quenched upon oxidation, while the fluorescence wavelength with an excitation wavelength of 480 nm emits light upon participation. For measurement of the amount of intracellular H_2_O_2_, the fluorescence intensity at 480 nm, *I*_480_, divided by that at 400 nm, *I*_400_, (= *I*_480_/*I*_400_) was normalized by the fluorescence intensity ratio at the beginning of the observation. That is, the variation, Δ*I*_480_/Δ*I*_400_, in HyPer7 from the initial state was evaluated. First, HyPer7 was expressed in the cells by transfection with Lipofectamine. Before using the HyPer7 plasmid, it was washed with ethanol (057‐00456, Fujifilm Wako Pure Chemical Corporation, OSA, JP). Dissolve the washed HyPer7 plasmid in reduced‐serum medium (31985062, Thermo Fisher Scientific, MA, US) and add Lipofectamine P3000 (L3000008, Thermo Fisher Scientific, MA, US). The prepared HyPer7 plasmid solution was sprayed on the cells and incubated (37°C, 5% CO_2_) for 1 day. After incubation, the medium was replaced, and the cells were incubated (37°C, 5% CO2) for an additional two days. During the experiment, the amount of medium in the dish was added to bring the volume of medium in the glass‐bottom dish to 4 mL. The glass‐bottom dishes containing HyPer7‐transfected cells were irradiated. Fluorescence changes of HyPer7 were observed in time‐lapses images during ultrasound irradiation. In the experiments using HyPer7, four independent experiments were conducted, each measuring the fluorescence intensity of 10 cells.

### 5.7 Evaluation of Intracellular ROS by Changing the Acoustic Pressure and Frequency

Cells with fluorescent probe or gene expression were irradiated with 6.5 MHz ultrasound. Experiments were conducted with different acoustic pressures and frequencies. In the experiments with different acoustic pressures, the acoustic pressure varied from 0 to 2 MPa at 1 MPa intervals. At an acoustic pressure exceeding 3 MPa, the LN transducer cracked during ultrasound irradiation, so experiments were conducted at an acoustic pressure of 2MPa or less. In experiments with different frequencies, 1.0 and 6.5 MHz, were used in the experiments with different frequencies. A 0 MHz condition was also provided as a control condition. Acoustic pressure was held constant at 2 MPa under all conditions. Ultrasound irradiation was continuous for 5 min in both experiments with varying acoustic pressure and frequency. Note that the PZT transducer for 1 MHz ultrasound generates heat immediately when operated at a similar acoustic pressure level as the LN transducer. Therefore, the irradiation time was set to 5 min to suppress the temperature rise of the PZT. During ultrasound irradiation, time‐lapse observation was conducted using a microscope to observe changes in the fluorescence intensity of the cells over time.

### 5.8 Evaluation of Intracellular ROS by Changing the Oxidative Stress Condition

Cells with fluorescent probe were subjected to four types of oxidative stress. In the 0 MHz ultrasound condition, cells were not irradiated, but were left standing when cells were observed with a microscope. Under the condition of 6.5 MHz ultrasound irradiation, the irradiation was continuous for 5 min at an acoustic pressure of 2 MPa. In the condition of ultrasound irradiation with NAC, the cells were pre‐incubated for 6 h in medium containing 5 mm NAC. After incubation, the cells were continuously irradiated with 6.5 MHz ultrasound at 2 MPa for 5 min. Under the thermal control condition, the temperature was controlled using a temperature‐controlled chamber (STXG‐UKX‐SET, Tokai Hit, JP). The temperature was set to 32°C. During ultrasound irradiation, time‐lapse observation was conducted using a microscope to observe changes in the fluorescence intensity of the cells over time.

### 5.9 Evaluation of the Percentage of Dead Cells by Ultrasound Irradiation

To evaluate the effect of ultrasound irradiation on cells, 4 conditions were prepared: 0 MHz ultrasound condition, 1 MHz ultrasound irradiation, 6.5 MHz ultrasound irradiation, and a thermal control condition. Under the 0 MHz ultrasound condition, the sample was left to stand for 5 min. Under ultrasound irradiation conditions, ultrasound was applied for 5 min at an acoustic pressure of 2 MPa across all frequencies. Under thermal control conditions, samples were heated at 32°C for 5 min. The samples were incubated (37°C, 5% CO_2_) after each treatment and stained at incubation times of 0, 6, and 24 h. The stained samples at each time were independent. Calcein‐AM (C396, Dojindo Laboratories, JP) was used to stain living cells. PI (P378, Dojindo Laboratories, JP) was used to stain dead cells. First, 10 µL each of PI and Calcein‐AM at a concentration of 1 mg/ml were diluted into 6 mL of culture medium. Cells were incubated (37°C, 5% CO_2_) for 30 min after treatment with the prepared dilution solution. After staining with PI and Calcein‐AM, cells were stained with diluted Hoechst solution to stain cell nuclei. Diluted Hoechst solution was prepared by diluting 10 ml of 1 mg/ml Hoechst 33342 solution (H342, Dojindo Laboratories, JP) with 5 mL of culture medium. After staining, the cells were observed. Only live and dead cells stained with Hoechst were counted.

### 5.10 Evaluation of Intracellular ROS by Ultrasound Irradiation in Spheroids

First, Spheroids were prepared using a well plate (MS‐9384U, Sumitomo Bakelite CO., Ltd., JP). HeLa cells (6 × 10^3^/well) were cultured for two days to form spheroids. The spheroids were embedded in collagen gel in glass‐bottom dishes. The collagen gel was prepared by mixing collagen acid solution (IPC‐50, Koken Co., Ltd., JP), Hank's balanced salt solution (H1641, Sigma‐Aldrich CO., LLC., US), and HEPES buffer (H3375, Sigma‐Aldrich CO., LLC., US) in an 8:1:1 ratio. After placing collagen and spheroids on the glass‐bottom dish, the spheroids were incubated for 30 min (37°C, 5% CO_2_) to fix them with collagen. After incubation, spheroids were stained with Calcein‐AM. Spheroids were incubated for 30 min (37°C, 5% CO_2_) for staining. After staining with Calcein‐AM, stained with OxiORANGE. At this step, the cells were incubated for 20 min (37°C, 5% CO_2_). After staining with OxiORANGE, the solution in the glass‐bottom dish was replaced with PBS (−) and observed under each condition. Under the 0 MHz ultrasound condition, observations were made without applying ultrasound. Under both conditions of 1 and 6.5 MHz ultrasound irradiation, irradiation was conducted at 2 MPa for 5 min. Under 6.5 MHz ultrasound irradiation with NAC, spheroids treated with 5 mm NAC for 6 h were irradiated with 6.5 MHz ultrasound at 2 MPa for 5 min. The spheroid was imaged from the upper half while being irradiated with ultrasound. By evaluating the changes in fluorescence intensity due to ROS generation through time‐series comparison of the obtained images as Z‐stacks. The pixel distribution histograms of brightness values were compared to evaluate changes in fluorescence intensity [73]. The pixel distribution of brightness values was obtained by aggregating the brightness values of pixels across the entire spheroid at each time. Changes in the fluorescence intensity of spheroids were observed due to shifts in pixel distribution. Additionally, colormap images were created to facilitate understanding of changes in spheroid images. The colormap images were obtained by converting the acquired spheroid images into grayscale images and applying the “Jet” color scheme as a false color. A calibration bar was added to the created colormap images to show that the same processing was applied in each condition.

### 5.11 Statistical Analysis

For the comparison of groups, variance (ANOVA) with Shaffer's method was used [74]. The ^*^ *p* < 0.05 or ^**^ *p* < 0.01 value was considered significant. Error bars and shaded error bands in the graphs indicate standard deviation. In addition, to calculate the effect size, linear regression analysis was used. The *R^2^* value was calculated as the effect size [75].

## Conflicts of Interest

The authors declare no conflicts of interest.

## Supporting information




**Supporting File**: advs74534‐sup‐0001‐SuppMat.docx.

## Data Availability

The data that support the findings of this study are available from the corresponding author upon reasonable request.
