# Supplementary material for: Intracellular Reactive Oxygen Species Generation Induced by High‐Frequency Ultrasound in Thickness Vibration Mode
Source: Adv Sci (Weinh). 2026 Feb 21;13(28):e19582. doi: 10.1002/advs.202519582 (PMC13185835; doi:10.1002/advs.202519582)
Supplement: Supplementary file 1 — Supporting File: advs74534‐sup‐0001‐SuppMat.docx. [file ADVS-13-e19582-s001.docx]

Supporting Information

Intracellular reactive oxygen species generation induced by high-frequency ultrasound in thickness vibration mode

Kotaro Fujishiro, Satoshi Okada, Filippo Rossi, Takahiro Kuchimaru, and Yuta Kurashina*


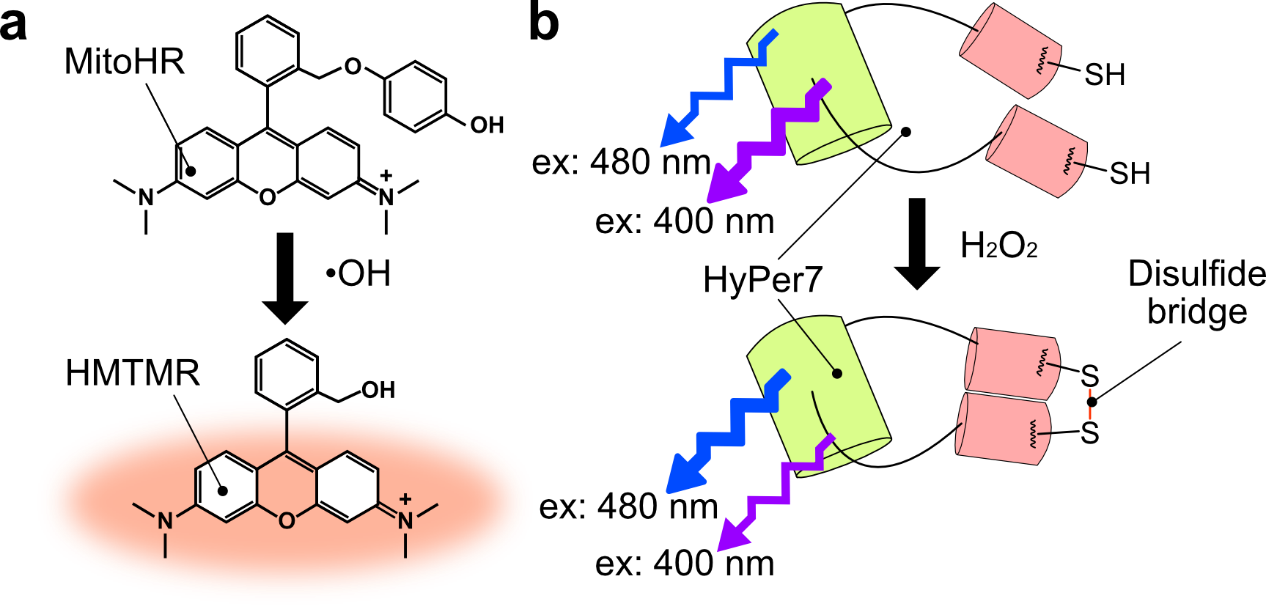


**Figure S1**. Fluorescent indicator and ROS reaction. (a,b) Visualization method of (a) •OH by OxiORANGE and (b) H_2_O_2_ by HyPer7.


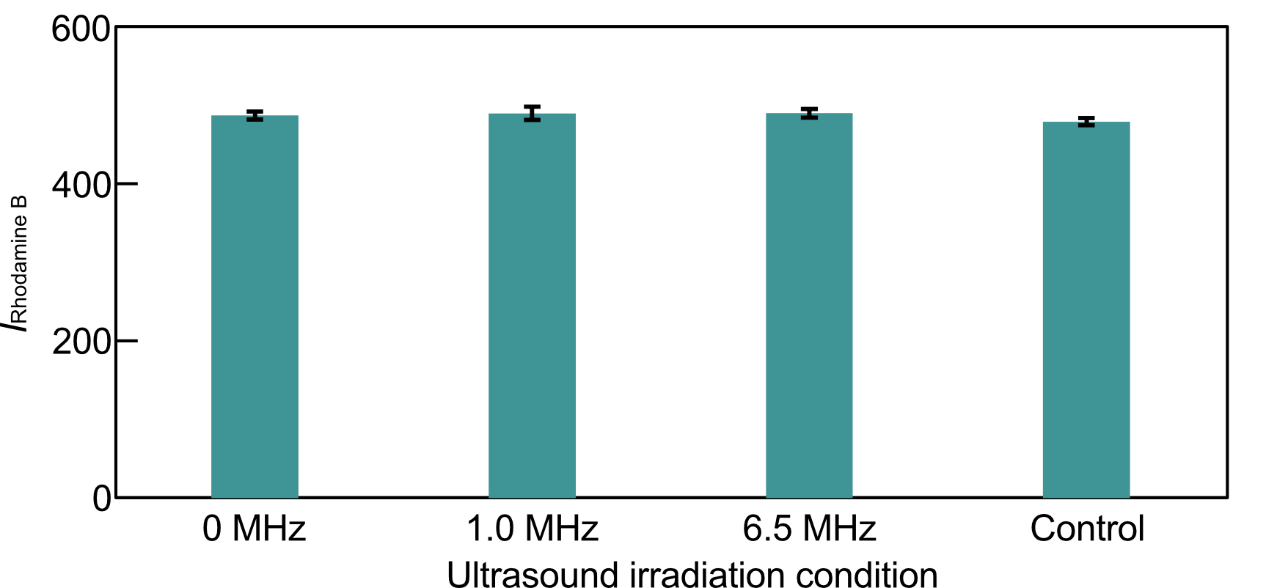


**Figure S2**. Fluorescence intensity of Rhodamine B, *I*_Rhodamine B_, in supernatant after ultrasonic irradiation. Measurements were taken to confirm that the fluorescent dye component of OxiORANGE did not react to ultrasound. The control condition was when 100 μM hydrogen peroxide was dispersed in a supernatant*.* Error bars: mean ± SD. *n* =3.

**
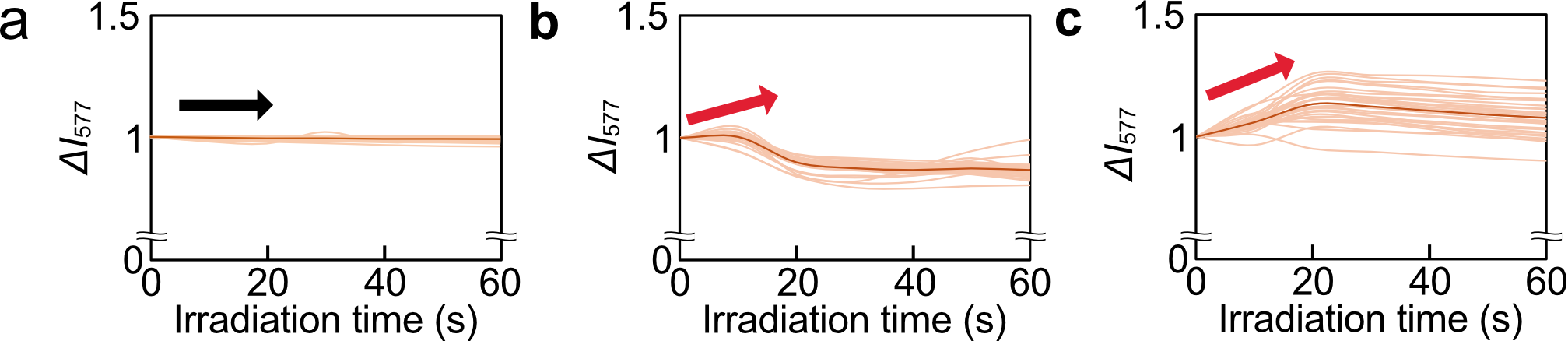
Figure S3**. Time-course graph of normalized OxiORANGE fluorescence intensity, *ΔI*_577_, during acoustic pressure changes at (a) 0, (b) 1, and (c) 2 MPa. The orange lines represented the fluorescence change for each of the 30 cells, and the brown lines represented the average value. The red and black arrows indicate an increase or decrease in fluorescence intensity. *n* =30.


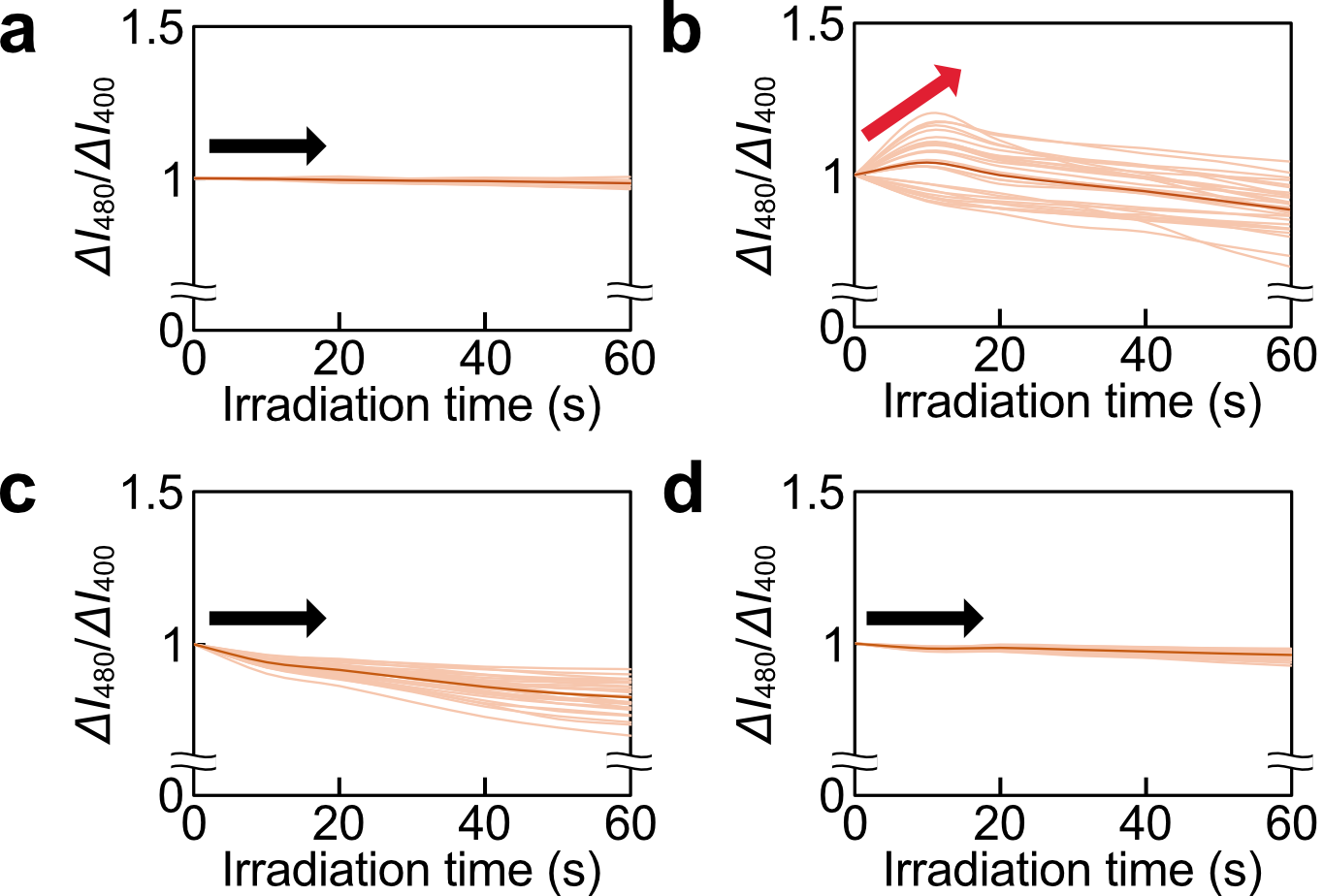


**Figure S4**. Time-course graph of normalized OxiORANGE fluorescence intensity, *ΔI*_577_, during oxidative stress conditions at (a) Control, (b) 6.5 MHz ultrasound, (c) 6.5 MHz ultrasound with NAC, and (d) Thermal control. Note that the sample was heated to 32 °C, which was the temperature increase caused by the ultrasound. The orange lines represented the fluorescence change for each of the 30 cells, and the brown lines represented the average value. The red and black arrows indicate an increase or decrease in fluorescence intensity. *n* =30.

**
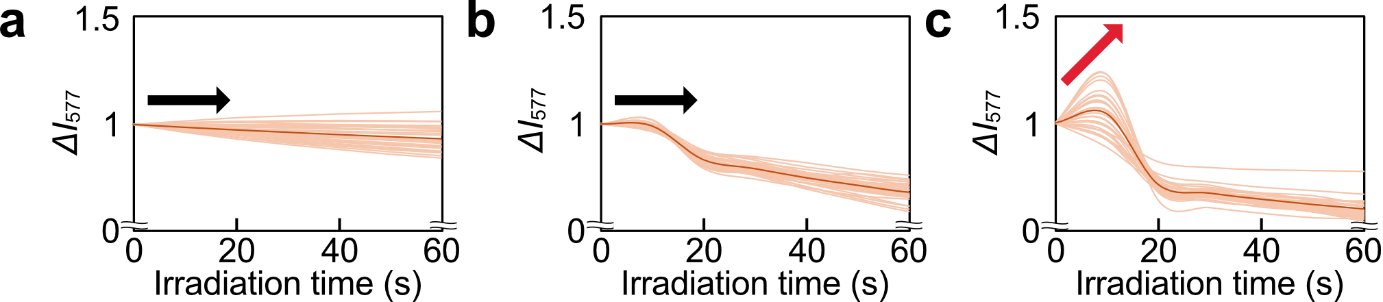
Figure S5**. Time-course graph of normalized OxiORANGE fluorescence intensity, *ΔI*_577_, during frequency changes at (a) 0, (b) 1, and (c) 6.5 MHz. The orange lines represented the fluorescence change for each of the 30 cells, and the brown lines represented the average value. The red and black arrows indicate an increase or decrease in fluorescence intensity. *n* =30.


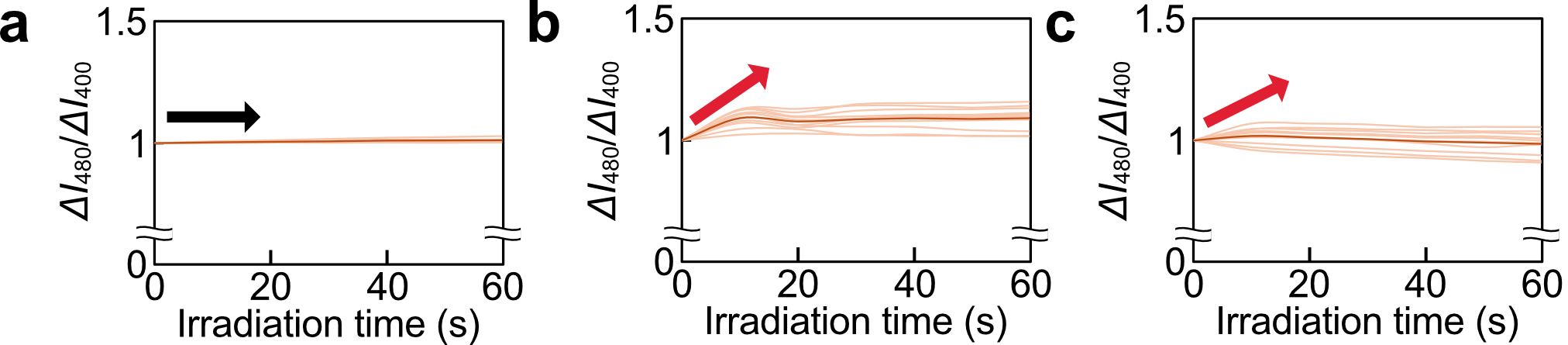


**Figure S6**. Time-course graph of normalized HyPer7 fluorescence intensity, *ΔI*_480_/*ΔI*_400_, during acoustic pressure changes at (a) 0, (b) 1, and (c) 2 MPa. The orange lines represented the fluorescence change for each of the 10 cells, and the brown lines represented the average value. The red and black arrows indicate an increase or decrease in fluorescence intensity. *n* =10.


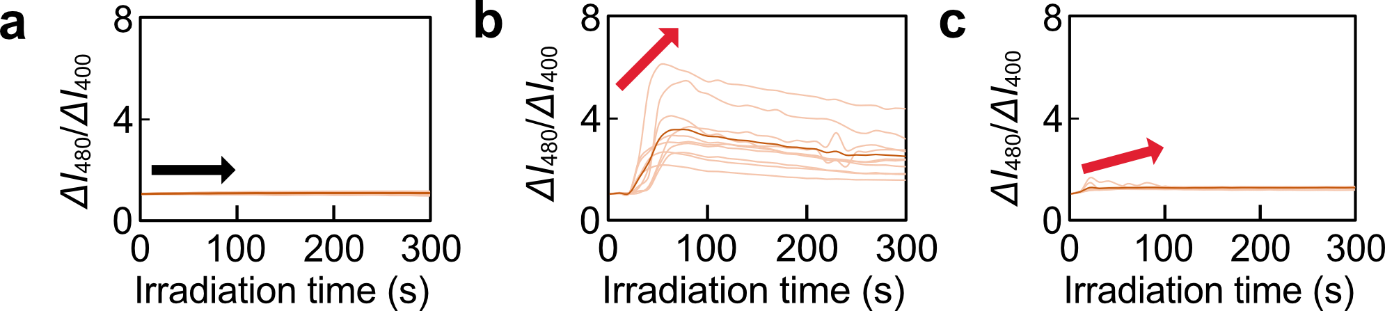
**Figure S7**. Time-course graph of normalized HyPer7 fluorescence intensity, *ΔI*_480_/*ΔI*_400_, during frequency changes at (a) 0, (b) 1, and (c) 6.5 MHz. The orange lines represented the fluorescence change for each of the 10 cells, and the brown lines represented the average value. The red and black arrows indicate an increase or decrease in fluorescence intensity. *n* =10.

| Components | (mg/L) | Components | (mg/L) |
| --- | --- | --- | --- |
| CaCl_2_ | 200.00 | Folic Acid | 1.00 |
| KCl | 400.00 | i-Inositol | 2.00 |
| MgSO_4_ | 98.00 | Niacinamide | 1.00 |
| NaCl | 6800.00 | Pyridoxal HCl | 1.00 |
| NaHCO_3_ | 2200.00 | Riboflavin | 0.10 |
| NaH_2_PO_4_ | 121.74 | Thiamine HCl | 1.00 |
| L-Arginine HCl | 126.00 | D-Glucose | 1000.00 |
| L-Cystine | 23.78 | Phenol Red | 10.00 |
| L-Glutamine | 292.00 |  |  |
| L-Histidine HCl H_2_O | 42.00 |  |  |
| L-Isoleucine | 52.00 |  |  |
| L-Leucine | 52.00 |  |  |
| L-Lysine HCl | 72.50 |  |  |
| L-Methionine | 15.00 |  |  |
| L-Phenylalanine | 32.00 |  |  |
| L-Threonine | 48.00 |  |  |
| L-Tryptophan | 10.00 |  |  |
| L-Tyrosine | 36.00 |  |  |
| L-Valine | 46.00 |  |  |
| D-1/2Ca Pantothenate | 1.00 |  |  |
| Choline Chloride | 1.00 |  |  |

**Table S1**. Composition of the medium used for cell culture.

**Table S2**. Composition of PBS (-).

| Components | (mg/L) |
| --- | --- |
| NaCl | 8181.60 |
| KCl | 201.29 |
| Na_2_HPO_4_ | 1419.60 |


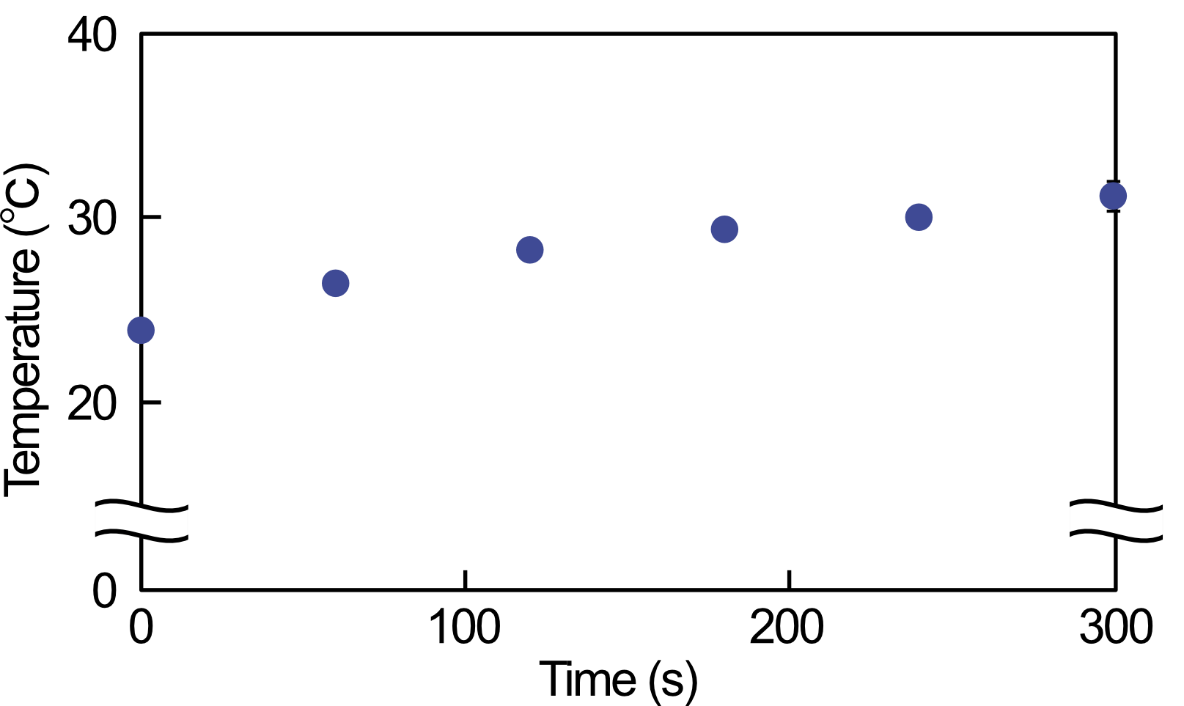
**Figure S8**. Temperature changes at the focus during 6.5 MHz ultrasound irradiation. Error bars: mean ± SD. *n* =3.
